# Cthrc1 deficiency aggravates wound healing and promotes cardiac rupture after myocardial infarction via non-canonical WNT5A signaling pathway

**DOI:** 10.7150/ijbs.79260

**Published:** 2023-02-13

**Authors:** Di Wang, Yaping Zhang, Tianbao Ye, Runlei Zhang, Lili Zhang, Dongmei Shi, Taixi Li, Guofang Xia, Kaifan Niu, Zhe Zhao, Yu Chen, Weijun Pan, Liang Liu, Xian Jin, Chengxing Shen

**Affiliations:** 1 Department of Cardiology, Shanghai Jiao Tong University Affiliated Sixth People's Hospital, 600 Yishan Road, Shanghai, China; 2 Department of General Practice, Qibao Community Health Service Center Affiliated to Shanghai University of Traditional Chinese Medicine, Shanghai, China; 3 Department of Geriatrics, Shanghai Jiao Tong University Affiliated Sixth People's Hospital, Shanghai, China; 4 Shanghai Institute of Nutrition and Health, Chinese Academy of Sciences, Shanghai, China

**Keywords:** CTHRC1, Fibroblast, Myocardial infarction, Cardiac repair, WNT5A.

## Abstract

Cardiac fibroblasts are crucial for scar formation and cardiac repair after myocardial infarction (MI). Collagen triple helix repeat containing 1 (CTHRC1), an extracellular matrix protein, is involved in the pathogenesis of vascular remodeling, bone formation, and tumor progression. However, the role and underlying mechanism of CTHRC1 in post-MI wound repair are not fully clear. Bioinformatics analysis demonstrated CTHRC1 up-regulation in cardiac fibroblasts after ischemic cardiac injury. Serum levels of CTHRC1 were increased in MI mice and CTHRC1 expression was up-regulated in cardiac fibroblasts after MI. *In vitro* results showed that the induction of CTHRC1 expression in cardiac fibroblasts was mediated by canonical TGFβ1-Smad2/3 signaling axis. Moreover, CTHRC1 improved wound healing and boosted cardiac fibroblast activation *in vitro*. Cthrc1 deficiency aggravated cardiac function and reduced collagen deposition as well as increased mortality attributable to cardiac rupture after MI. Consistent with above phenotypes, reduced the levels of myocardial CD31, α-smooth muscle actin, collagen I, and collagen III was observed, whereas myocardial expression of matrix metalloproteinase 2 and matrix metalloproteinase 9 were increased in Cthrc1 knockout mice post-MI. Above effects could be partly reversed by rCTHRC1 protein or rWNT5A protein. Our study indicates that cardiac fibroblast-derived, canonical TGFβ1-Smad2/3-dependent CTHRC1 could improve wound repair and prevent cardiac rupture after MI via selectively activating non-canonical WNT5A-PCP signaling pathway.

## Introduction

Acute myocardial infarction (AMI) has been regarded as the most severe clinical manifestation of coronary heart disease and remains the leading cause of morbidity and mortality worldwide 1, 2, in spite of substantial improvements in clinical prognosis over the past decade due to the optimal medical treatment and the extensive use of percutaneous coronary intervention as well as coronary artery bypass graft surgery. Patients who survived MI still suffer from risk of heart failure because of excessive inflammation, impaired wound healing and deformed scar formation, as well as enhanced cell loss and contractile dysfunction, consequently facilitating infarct expansion, cardiac rupture, and adverse remolding 3. Therefore, it is of importance to identify novel potential therapeutic targets to alleviate inflammation and promote wound repair after MI.

As is known to all, the reparative and remodeling process after MI can be separated into three distinct phases: the inflammatory phase (3 hours to 3 days), the proliferative phase (3 days to 14 days) and the scar stabilization and maturation phase (14 days to 2 months) 4. In the dynamic changed environment of the ischemic heart, cardiac fibroblasts display a functional diversity which may reflect their phenotypic heterogeneity and therefore contribute essentially to wound healing after MI. During the inflammatory phase of cardiac repair, pro-inflammatory cardiac fibroblasts can secrete multiple cytokines and chemokines to recruit and stimulate leukocytes, and release matrix metalloproteinases (MMPs) to facilitate extracellular matrix degradation as well as release of pro-inflammatory matrix fragments. Removal of deribs from dead cells in the infarcted heart activates anti-inflammatory signals, thereby resulting in resolution of inflammation and transformation into the proliferative phase of cardiac repair. Cardiac fibroblasts migrate as well as undergo myofibroblast differentiation with incorporation of α-smooth muscle actin (α-SMA) into stress fibers and activated myofibroblasts deposit an abundance of extracellular matrix proteins to prevent cardiac rupture. During scar stabilization and maturation, cardiac fibroblasts disassemble α-SMA-decorated stress fibers and secrete matrix-crosslinking enzymes, which can further promote extracellular matrix crosslinking and remolding. Up to now, the molecular basis for the phenotypic transformation of cardiac fibroblasts in the phase of cardiac repair remains unclear. Thus, understanding the endogenous mechanisms of cardiac fibroblast phenotypic transition may help discover novel promising therapeutic targets post-MI.

To identify the functional candidate genes potentially related to cardiac fibroblast activation after ischemic cardiac injury, we resorted to the multicellular transcriptional dataset 5 downloaded from the Gene Expression Omnibus (GEO) database and a prior published single-cell RNA sequencing data 6 to screen differentially expressed genes (DEGs) in cardiac fibroblasts post-MI. The up-regulated molecule collagen triple helix repeat containing 1 (CTHRC1) attracted our attention owing to its high conservatism and association with vascular remolding, which is worthy of further investigation in ischemic heart disease. CTHRC1, a secreted extracellular matrix glycoprotein that is highly conserved from lower chordates to mammals 7, plays an essential role in biological functions. Prior studies showed that CTHRC1 participated in varieties of physiological as well as pathological processes, containing vascular remodeling 7-10, bone formation 11-15, developmental morphogenesis 16-20, rheumatoid arthritis 21-24, glucose and lipid metabolism 25-27, as well as organ fibrosis (such as dermal 28-30, lung 31-33, and liver fibrosis 34, 35). A rapidly growing number of studies have demonstrated that CTHRC1 was highly expressed in a wide range of human solid tumors and functionally related to tumor cell proliferation, migration, invasion, metastasis, as well as tumor angiogenesis 36-39. More recently, Adrián et al. 40 used single-cell RNA sequencing to identify the new CTHRC1^+^ sub-population of cardiac fibroblasts in post-MI mice, which localized into the scar and was characterized by profibrotic action. They showed that the absence of Cthrc1 induced lethality caused by cardiac rupture in mice after MI. However, the precise role and potential underlying mechanisms of CTHRC1 in wound healing after MI are not fully elucidated. Therefore, this study aimed to explore the effect of CTHRC1 on post-infarction cardiac repair and its underlying molecular mechanisms.

## Materials and Methods

An expanded and detailed materials and methods section of this study is available in the Online Data Supplement.

### Ethics statement

All experiments involving animals were conducted according to the ethical policies and procedures approved by the ethics committee of Shanghai Jiao Tong University Affiliated Sixth People's Hospital, China (Approval no. DWLL2022-0462).

### Statistical Analysis

All values are presented as the mean±standard error of the mean (SEM) of independent experiments or independent samples with given n sizes. Statistical analysis was performed with GraphPad Prism 7.0 (Graph Pad Prism Software, Inc, San Diego, CA). Detailed statistical analysis is provided in the Online Data Supplement.

## Results

### Serum levels of CTHRC1 were elevated in AMI patients and in MI mice and CTHRC1 expression was increased in cardiac fibroblasts after MI

**Figure 1:**
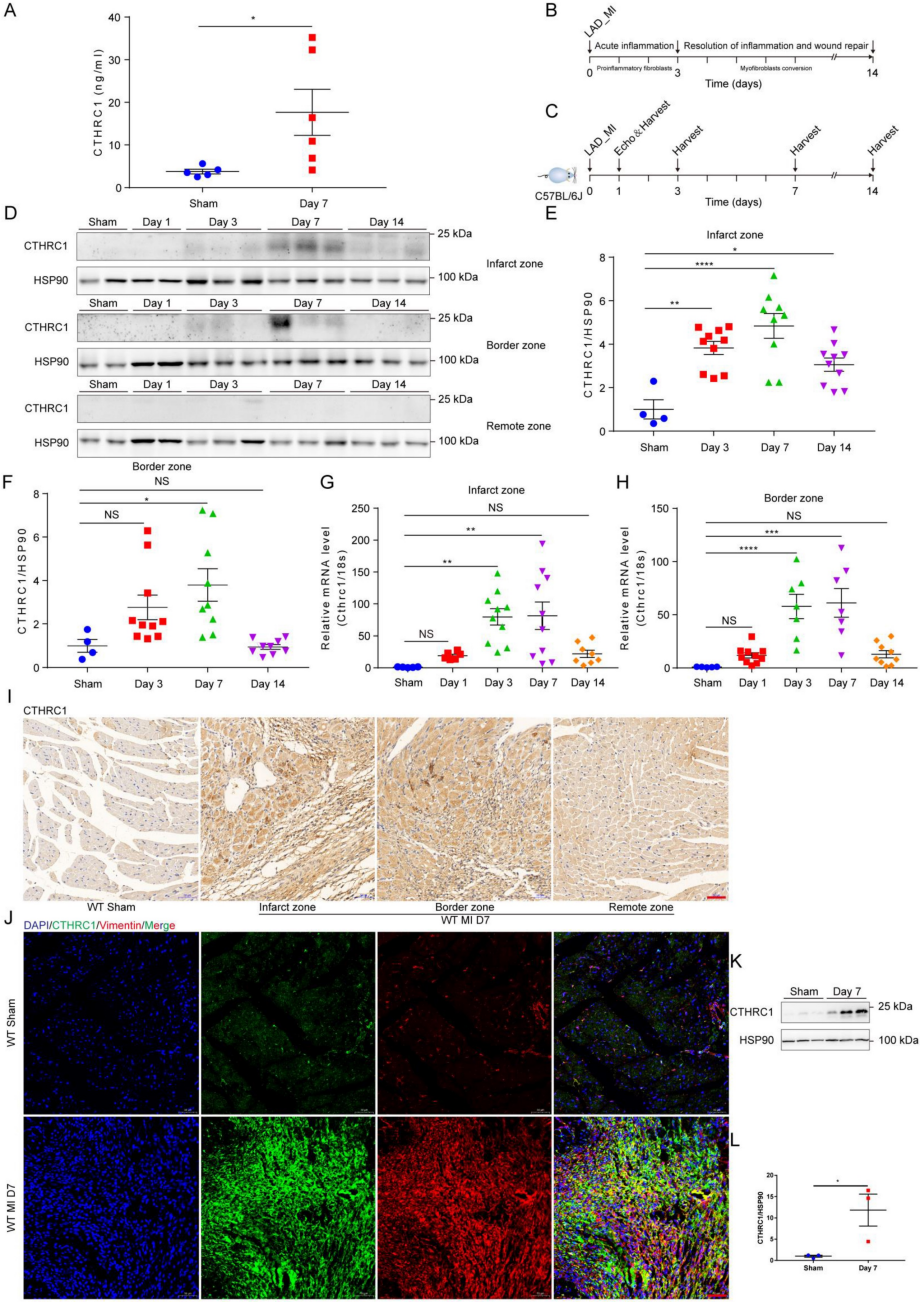
** Serum levels of CTHRC1 were elevated in MI mice and CTHRC1 expression was increased in cardiac fibroblasts after MI. (A)** Serum CTHRC1 levels in MI mice at day 7 after MI were measured by ELISA (n=5-6). **(B)** Two crucial phases of cardiac repair after MI. **(C)** Timeline of the experimental design for Figure 1. **(D)** CTHRC1 expression was analyzed by western blotting in the infarct zone, in the border zone, as well as in the remote zone of WT hearts at different time points post- MI. **(E and F)** Quantitative analyses of western blotting band intensity in D (n=4-10). **(G and H)** CTHRC1 mRNA expression was determined in the infarct and the border areas of WT MI-operated hearts and sham controls at different time points post-MI (n=5-10). **(I)** Representative immunohistochemistry analyses of CTHRC1 in both sham-operated and WT post-MI mice, including the infarct zone, the border zone, as well as the remote zone (bar=50 μm). **(J)** Immunofluorescence co-staining for CTHRC1 with vimentin in WT hearts at day 7 after MI (bar=50 μm). **(K and L)** Primary cardiac fibroblasts were isolated from WT hearts at day 7 post-MI, and CTHRC1 protein expression was examined and quantified (n=3). Data are presented as mean±SEM. *P<0.05. **P<0.01. ***P<0.001. ****P<0.0001. NS: not significant. Data were analyzed using one-way ANOVA followed by Bonferroni multiple comparison test **(E-H)** and unpaired two-tailed Student's t test **(A and L)**.

To identify the novel candidate genes potentially correlated with the phenotypic transition of cardiac fibroblasts after MI, the multicellular transcriptional dataset 5 GSE95755 downloaded from the GEO database was analyzed. DEGs were presented by a heat map (Figure **S1A**) and a volcano pot (Figure **S1B**). Selected up-regulated DEGs showed that CTHRC1 expression was significantly up-regulated in cardiac fibroblasts post-MI (Figure **S1C**). Furthermore, the prior published single-cell RNA sequencing 6 performed on four main cardiac cell types, including cardiac fibroblasts, cardiomyocytes, endothelial cells, as well as macrophages under homeostatic and ischemic conditions, which revealed cardiac fibroblasts highly expressed CTHRC1 3 days post-ischemia/reperfusion, in comparison with other cardiac cell populations (Figure **S1D**). To evaluate the clinical relevance of CTHRC1 in human AMI, serum levels of CTHRC1 were measured in AMI patients (n=40) compared to healthy people (n=40). We found that serum CTHRC1 levels of human were increased at day 7 after MI (Figure **S2**), which is consistent with those in MI mice (Figure **1A**). As depicted in Figure **1B**, early inflammatory phase after MI is characterized by massive cellular infiltration and tissue digestion, lasting up to 3 days in mice, subsequently followed by a phase of active resolution of inflammation and wound repair with myofibroblast conversion (lasting≈3 days-14 days in mice) 4. To clarify the function of CTHRC1 in MI, we first investigated CTHRC1 expression levels in the myocardium at different time points after MI (Figure **1C**). In comparison with sham controls, CTHRC1 increased after MI, peaking at day 7 post-MI (Figure **1D**-**1H**). Moreover, CTHRC1 expression were significantly higher in the infarct and border zones in comparison with the remote zone in WT mice (Figure **1D**). Consistently, immunohistochemistry analyses of heart tissue samples confirmed that CTHRC1 was predominantly expressed in the infarct as well as border areas in conformity to the localization of vimentin, with low expression in the remote area (Figure **1I** and** S3A**). Western blotting results using the isolated primary cardiac fibroblasts and cardiomyocytes from WT mice with or without MI further confirmed that CTHRC1 was mainly expressed in cardiac fibroblasts in the post-MI heart tissue (Figure **1K**,** 1L**, and** S3B**). Immunofluorescence co-localization for CTHRC1 with vimentin, cTnI, and CD31 in infarcted heart tissue from WT mice collected at day 7 post-MI revealed the enrichment of CTHRC1 in cardiac fibroblasts (Figure **1J**), the low expression in endothelial cells (Figure** S3C**) but not in cardiomyocytes (Figure** S3D**). These data elucidate CTHRC1 enrichment in cardiac fibroblasts in the infarcted tissue during the proliferative phase of MI, implying a potential role of CTHRC1 in modulating wound healing and cardiac fibroblast activation post-MI.

### Induction of CTHRC1 by canonical TGFβ1-Smad2/3 signaling axis in cardiac fibroblasts

**Figure 2:**
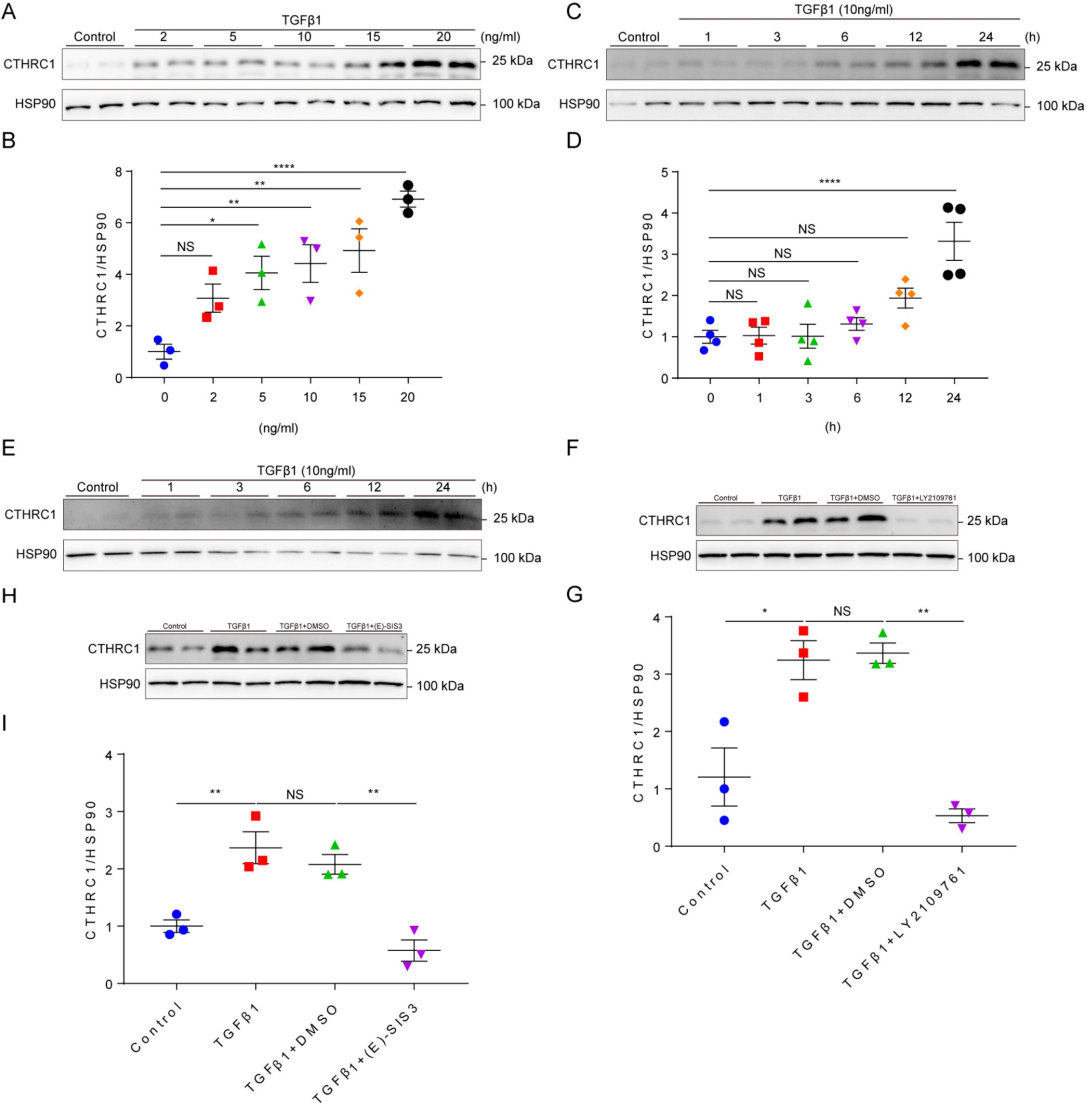
** Induction of CTHRC1 by canonical TGFβ1-Smad2/3 signaling axis in cardiac fibroblasts. (A and B)** Immunoblotting of primary cardiac fibroblasts stimulated with serial concentrations of TGFβ1 for 24 h (n=3).** (C and D)** CTHRC1 up-regulation induced by TGFβ1 (10 ng/ml) in primary cardiac fibroblasts isolated from WT hearts at different time points (n=4).** (E)** CTHRC1 up-regulation induced by TGFβ1 (10 ng/ml) in primary cardiac fibroblasts isolated from neonatal mouse hearts at different time points. **(F-I)** Immunoblot analyses of primary cardiac fibroblast treated with TGFβ1 (10 ng/ml) for 24 h, in the presence of LY2109761 **(10 μM, F and G)** or **(E)**-SIS3 **(10 μM, H and I)** (n=3). Data are presented as mean±SEM. *P<0.05. **P<0.01. ****P<0.0001. NS: not significant. Data in **B**, **D**, **G**, and **I** were analyzed using one-way ANOVA followed by Bonferroni multiple comparison test.

It is well established that TGF-β1 is the primary factor that activate fibroblasts and drives fibrosis acting through a canonical Smad signaling pathway that involves Smad4 binding to the phosphorylation of Smad2 and Smad3 by the TGFβ receptor I/II 41. Since CTHRC1 was predominantly expressed in cardiac fibroblasts, we then explored whether TGF-β1 can induce CTHRC1 expression in cardiac fibroblasts *in vitro*. Primary cardiac fibroblasts from WT mice were treated with serial concentrations of TGFβ1 and stimulated by TGF-β1 at different time points. The results showed that CTHRC1 was induced by TGFβ1 in a dose-dependent (Figure **2A** and** 2B**) and in a time-dependent manner (Figure **2C** and **2D**), which was also found in neonatal mouse primary cardiac fibroblasts treated with TGFβ1 (Figure **2E**). LY2109761, the specific inhibitor for TGFβ receptor I/II or E-SIS3, the specific inhibitor for the phosphorylation of Smad3 were used to interrupt the canonical TGFβ1 signaling pathway. As expected, TGFβ1 induced CTHRC1 up-regulation was inhibited in primary cardiac fibroblasts (Figure **2F** through **2I**). In summary, these data indicate the induction of CTHRC1 by canonical TGFβ1-Smad2/3 signaling pathway in cardiac fibroblasts.

### CTHRC1 improved wound healing and promoted cardiac fibroblast activation *in vitro*

**Figure 3:**
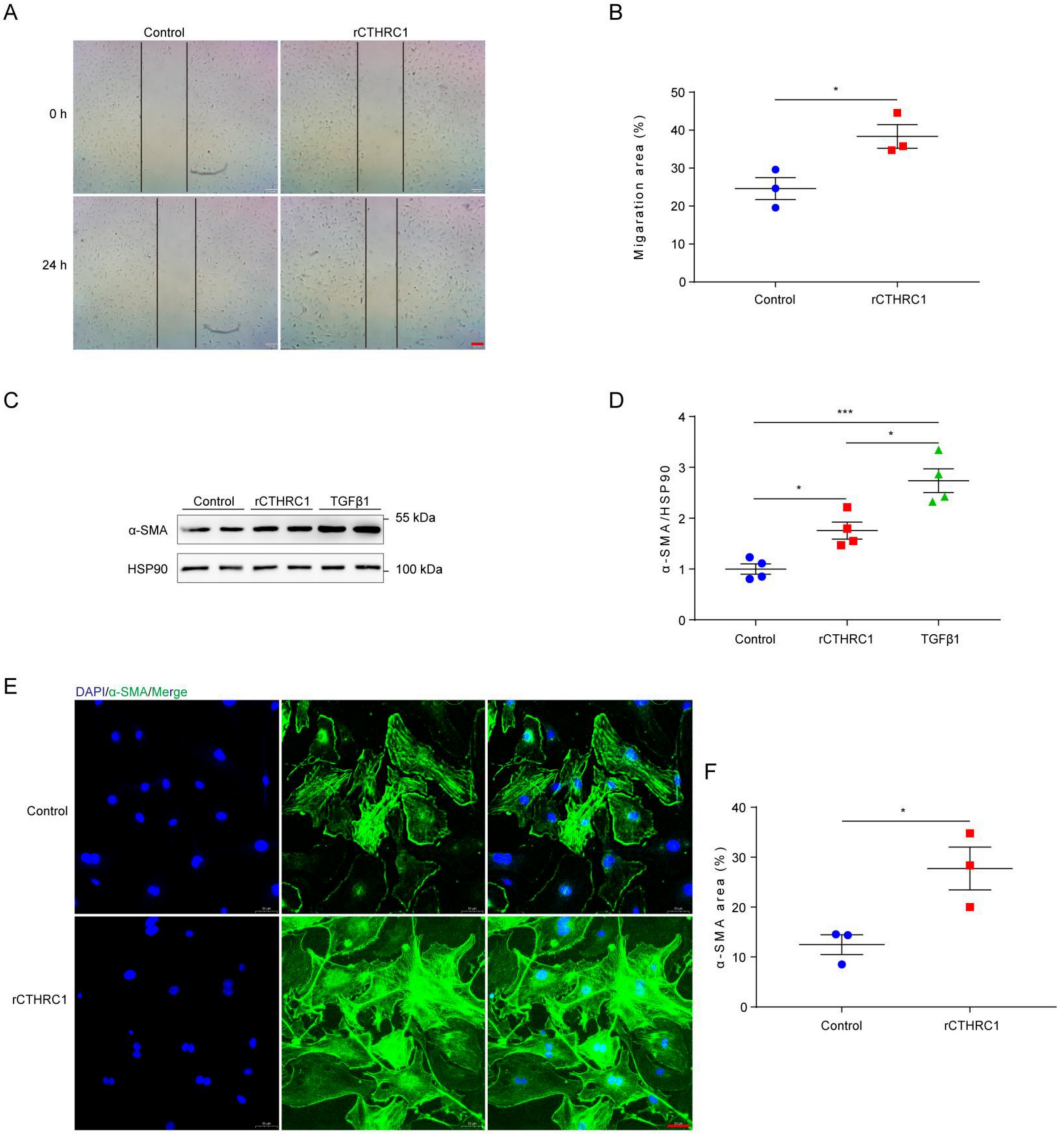
** CTHRC1 improved wound healing and promoted cardiac fibroblast activation *in vitro***.** (A and B)** The migratory capacity of cardiac fibroblasts isolated from WT hearts was assessed by the covering area of the scratch after 24 h (n=3, bar=200 μm). **(C and D)** α-SMA expression was detected by western blotting in primary cardiac fibroblasts following stimulation with rCTHRC1 (1 μg/ml) or TGFβ1 (10ng/ml) for 24h (n=4). **(E and F)** α-SMA immunofluorescence staining of primary cardiac fibroblasts treated with rCTHRC1 (1 μg/ml) for 24h was used to evaluate cardiac fibroblast activation (n=3, bar=50 μm). Data are presented as mean±SEM. *P<0.05. ***P<0.001. NS: not significant. Data were analyzed using one-way ANOVA followed by Bonferroni multiple comparison test **(D)** and unpaired two-tailed Student's t test **(B and F)**.

In view of CTHRC1 levels was increased in cardiac fibroblasts, we further investigated the effect of CTHRC1 on cardiac fibroblast migration and activation *in vitro*. Firstly, the impact of CTHRC1 on cardiac fibroblast migration was examined using an *in vitro* wound healing model. Compared with untreated primary cardiac fibroblasts, treatment of primary cardiac fibroblasts with 1 μg/mL of rCTHRC1 protein increased their migratory capacity, as evaluated by the covered area at 24 h after the initial scratch (Figure **3A** and **3B**). Stimulation with rCTHRC1 protein resulted in a significant up-regulation of α-SMA expression (Figure **3C** and **3D**). Immunofluorescence staining further confirmed that rCTHRC1 protein induced α-SMA expression in the primary cardiac fibroblasts (Figure **3E** and **3F**). To summarize, these results indicate that CTHRC1 can improve wound healing and promote cardiac fibroblast *activation in vitro*.

### Cthrc1 deficiency aggravated cardiac function and exacerbated MI-induced cardiac rupture as well as reduced collagen-rich scar formation after MI

**Figure 4:**
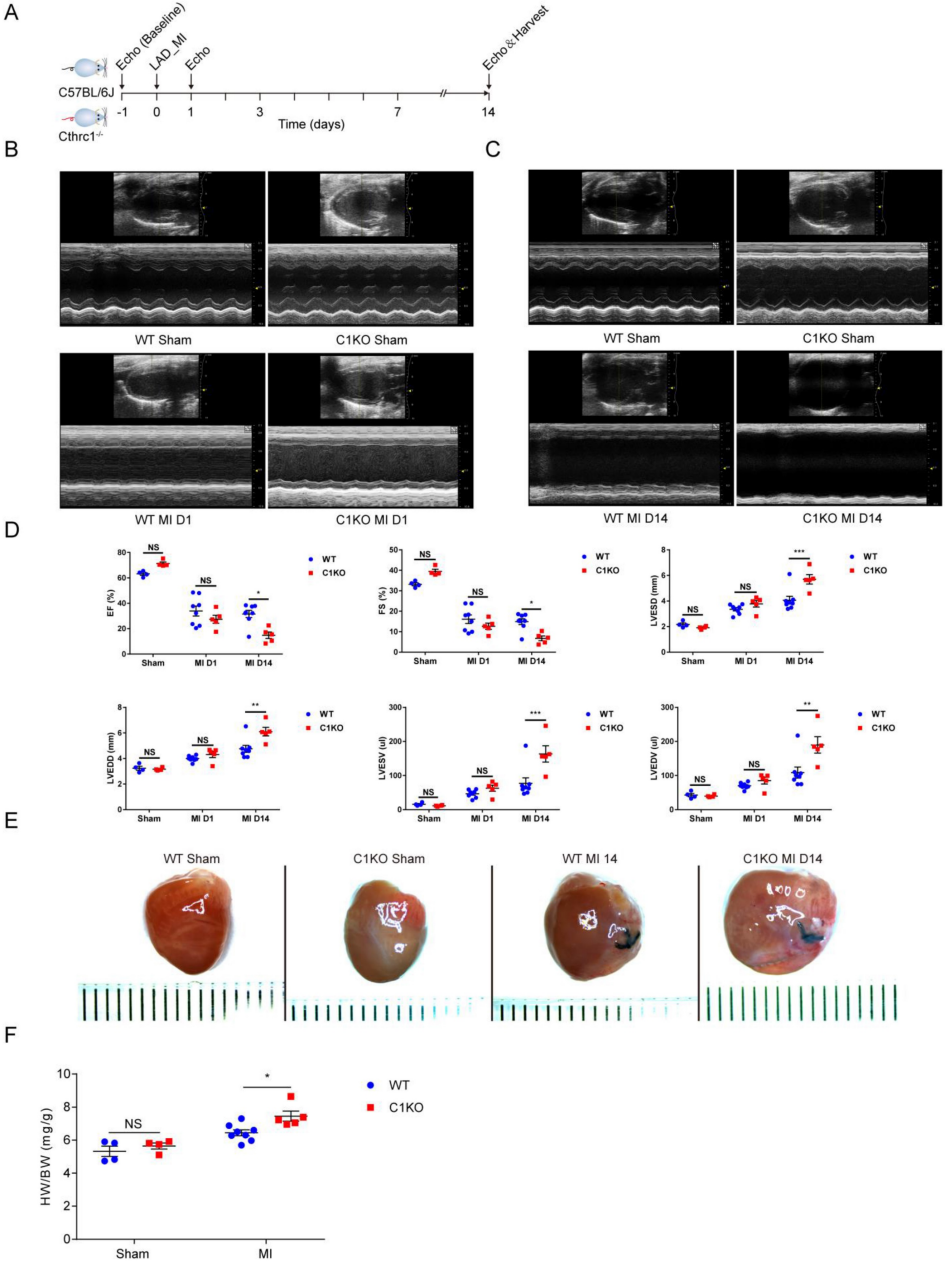
** Cthrc1 deficiency aggravated cardiac function after MI**.** (A)** Timeline of the experimental design for **Figure 4** and **5**. **(B and C)** Representative echocardiograms obtained from WT and C1KO mice at day 1 **(B)** and day 14 **(C)** post-MI or sham operation. **(D)** Echocardiographic assessment of cardiac function in WT and C1KO mice at day 1 and day 14 post-MI or sham surgery (n=4-8). **(E)** Representative macroscopic view of heart from MI-operated mice at day 14 after MI.** (F)** Heart weight to body weight ratio (HW/BW) in WT and C1KO mice subjected to MI for 14 days were analyzed (n=4-8). Data are presented as mean±SEM. *P<0.05. **P<0.01. ***P<0.001. NS: not significant. Data in **D** and **F** were compared using two-way ANOVA followed by Bonferroni multiple comparison test.

To explore the *in vivo* role of CTHRC1, we first examined whether Cthrc1 deficiency would impact cardiac function after MI (Figure **4A**). C1KO and WT male mice at 8-10 weeks of age were subjected to a permanent ligation of the LAD. Echocardiographic parameters measured in C1KO mice showed that Cthrc1 deficiency did not affect cardiac function in comparison with that in WT mice at baseline (Figure **S9**). Cardiac function was assessed at day 1 and day 14 post-MI. No significant differences in cardiac function between CIKO mice and WT mice were observed at day 1 after MI (Figure **4B** and **4D**), but EF, FS, LVESV, LVEDV, LVESD, and LVEDD were significantly exacerbated in survived C1KO mice compared with survived WT mice at day 14 post-MI (Figure **4C** and **4D**). TTC staining and masson's trichrome staining performed on serial heart cross sections demonstrated a similar increase of infarct size between WT and C1KO mice at day 7 after MI (Figure **S4A, 5D,** and** 5E**). Moreover, the ratio of heart weight to body weight at day 14 after MI was significantly higher in C1KO mice than that in WT mice (Figure **4E** and **4F**), suggesting their compromised cardiac function compared with that in WT mice.

**Figure 5:**
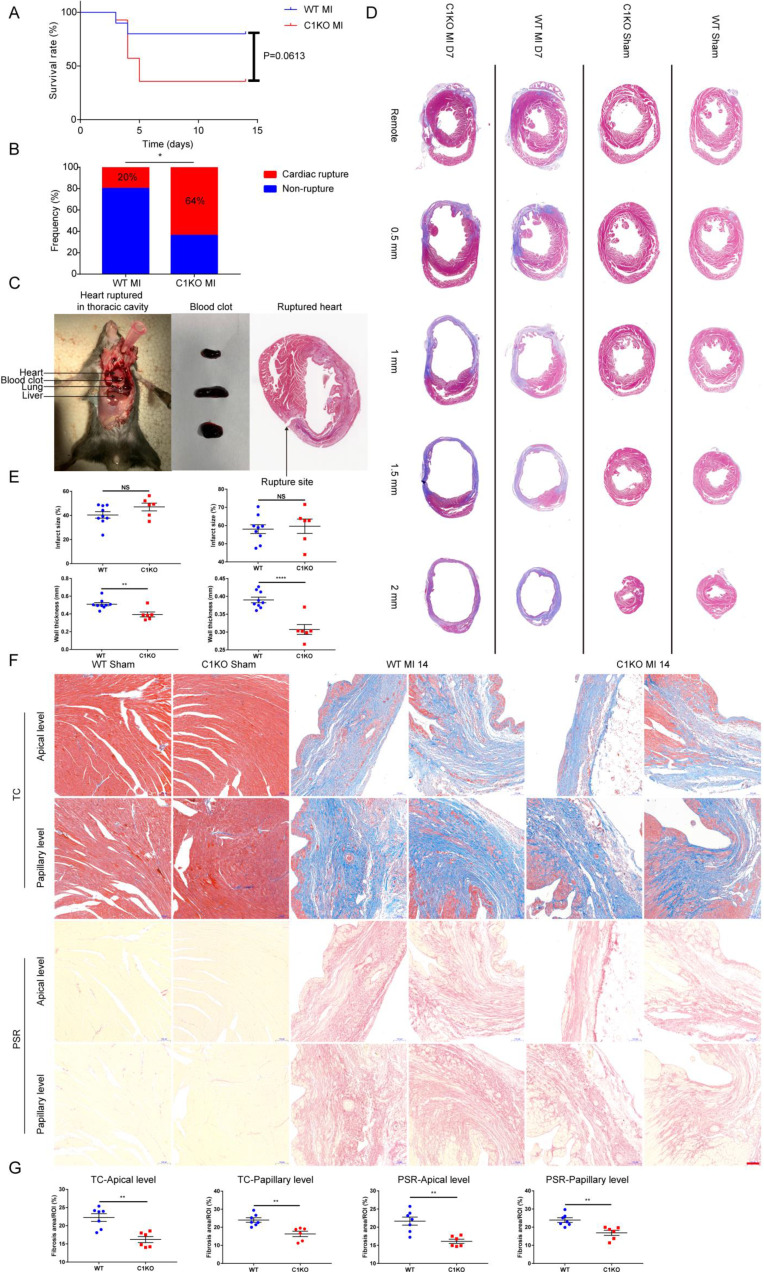
** Ablation of Cthrc1 exacerbated MI-induced cardiac rupture and reduced collagen-rich scar formation after MI**.** (A)** Kaplan-Meier survival analysis of mice at different time points after MI. **(B)** Total cardiac rupture frequency in WT (n=10) and C1KO (n=14) at day 14 after MI. **(C)** Representative macroscopic view of a hemothorax **(left)**, the presence of blood clots **(middle)** in the chest cavity and/or in the pericardium, and masson's trichrome staining **(right)** of cardiac rupture in C1KO mice at day 5 post-MI. **(D)** Masson's trichrome staining performed on serial heart transverse sections obtained from WT and C1KO mice at day 7 after LAD ligation. **(E)** Quantitative assessment of infarct size as well as wall thickness at day 7 after MI in WT and C1KO mice (n=6-9). **(F)** Masson's trichrome (TC) and picrosirius red (PSR) staining performed on heart cross sections at apical as well as papillary level to examine collagen density in the scar of WT and C1KO mice at day 14 after MI. **(G)** Quantification of fibrotic areas at day 14 after MI in WT and C1KO mice (n=6-7). Data are presented as mean±SEM. *P<0.05. **P<0.01. ****P<0.0001. NS: not significant. Survival rate was calculated by the Kaplan-Meier method and analyzed using log-rank test **(A)**. Data in **B** were compared using χ^2^ test. Data were analyzed by unpaired two-tailed Student's t test **(E and G)**.

Next, we investigated the influence of CTHRC1 on the post-MI survival. We found that the survival rate in C1KO mice was lower than that in WT mice within day 14 after MI (36% vs 80%; P=0.0613; Figure **5A**). The rate of cardiac rupture was significantly higher in C1KO mice, as compared with WT mice (64% vs 20%; P=0.0318; Figure **5B** and **5C**). Similarly, masson's trichrome staining performed on sequential heart transverse sections demonstrated Cthrc1 deficiency did not affect infarct size between WT and C1KO mice at day 7 after MI, but resulted in decreased wall thickness in the infarct zone in comparison with that in WT mice on day 7 after MI (Figure **5D** and** 5E**). To further clarify mechanism underlying the increased incidence of cardiac rupture post-MI in C1KO mice, collagen density in the infarct zone was evaluated by masson's trichrome staining and picrosirius red staining performed on cross sections of heart at apical as well as papillary level. The results demonstrated that collagen volume fraction in the infarct zone was significantly lower in C1KO hearts than that in WT hearts at day 14 post-MI (Figure **5F** and** 5G**). However, collagen density in the remote area was similar between WT and C1KO mice (Figure **S5A** through **S5C**). To conclude, this finding of higher risk of cardiac rupture in C1KO mice post-MI may be contributed to reduced collagen-rich scar formation in C1KO hearts.

**Figure 6:**
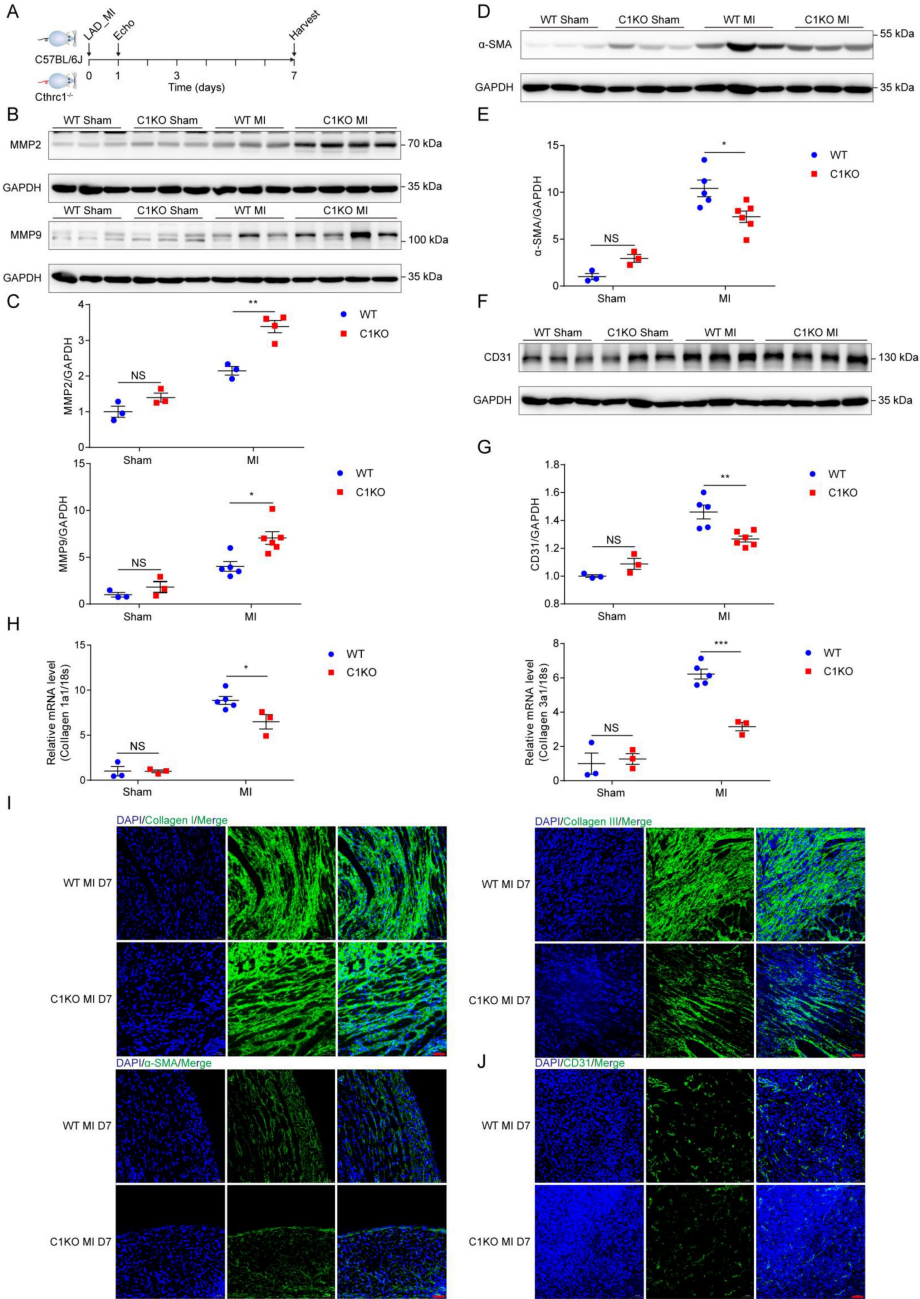
** Absence of Cthrc1 impaired wound repair after MI**.** (A)** Timeline of the experimental design for **Figure 6-7**. **(B and C)** Representative immunoblots **(B)** and summarized data **(C)** of MMP2 as well as MMP9 expression in the infarct areas of WT and C1KO hearts at day 7 after MI or post-sham operation (n=3-6). **(D and F)** α-SMA **(D)** and CD31 **(F)** protein levels were detected in the infarcted heart tissues from WT and C1KO mice after sham operation or day 7 post-MI. **(E and G)** Quantified data of western blotting band intensity in** D** and** F** (n=3-6).** (H)** Gene expression of collagen I and collagen III were analyzed by qRT-PCR in the infarct zones of WT and C1KO hearts at day 7 after MI (n=3-5). **(I and J)** Immunofluorescence analyses of collagen I, collagen III, and α-SMA **(I)** as well as CD31 **(J)** in the scar tissues obtained from WT and C1KO mice. Data are presented as mean±SEM. *P<0.05. **P<0.01. ***P<0.01. NS: not significant. Data in **C**, **E**, **G**, and **H** were compared using two-way ANOVA followed by Bonferroni multiple comparison test.

To explore the mechanism by which Cthrc1 deficiency promotes cardiac rupture, biomarkers for wound repair in the early phase of MI were analyzed (Figure **6A**). A variety of evidence supports the importance of MMP2 and MMP9 in cardiac repair after MI via breaking down collagens 42-44. Western blotting analysis demonstrated up-regulated MMP2 as well as MMP9 expression in the infarct area of C1KO hearts at day 7 post-MI, when compared with WT mice (Figure **6B** and **6C**). During the proliferative phase of cardiac repair, cardiac fibroblasts undergo myofibroblast transformation, incorporating α-SMA into stress fibers and activated myofibroblasts produce extracellular matrix proteins such as collagen I and collagen III, which boosts granulation tissue formation with greater tensile strength as well as more stress fibers to prevent post-infarction cardiac rupture 3. As expected, significantly reduced α-SMA protein levels (Figure **6D** and **6E**) as well as decreased gene expression of collagen I and collagen III (Figure **6H**) were observed in C1KO hearts at day 7 after MI, as compared with WT mice. Moreover, less extracellular matrix proteins deposition was evidenced in C1KO mice, as reflected by immunofluorescence staining of collagen I as well as collagen III on day 7 after MI (Figure **6I**). Less α-SMA^+^ myofibroblasts deposition was also evidenced in the infarct area of C1KO hearts (Figure **6I**). Post-infarction angiogenesis has been demonstrated to be essential for cardiac repair post-MI 3. In line with this, immunoblot analysis revealed that protein levels of CD31 were significantly lower in C1KO mice than that in WT control mice at day 7 after MI (Figure **6F** and **6G**). In addition, angiogenesis level determined by immunofluorescence staining of CD31 was lower at day 7 post-MI in C1KO mice (Figure **6J**). These results further suggest retarded cardiac repair post-MI may contribute to the higher cardiac rupture incidence in C1KO mice.

### CTHRC1 improved cardiac repair after MI via selectively activating non-canonical WNT5A-PCP signaling pathway

**Figure 7:**
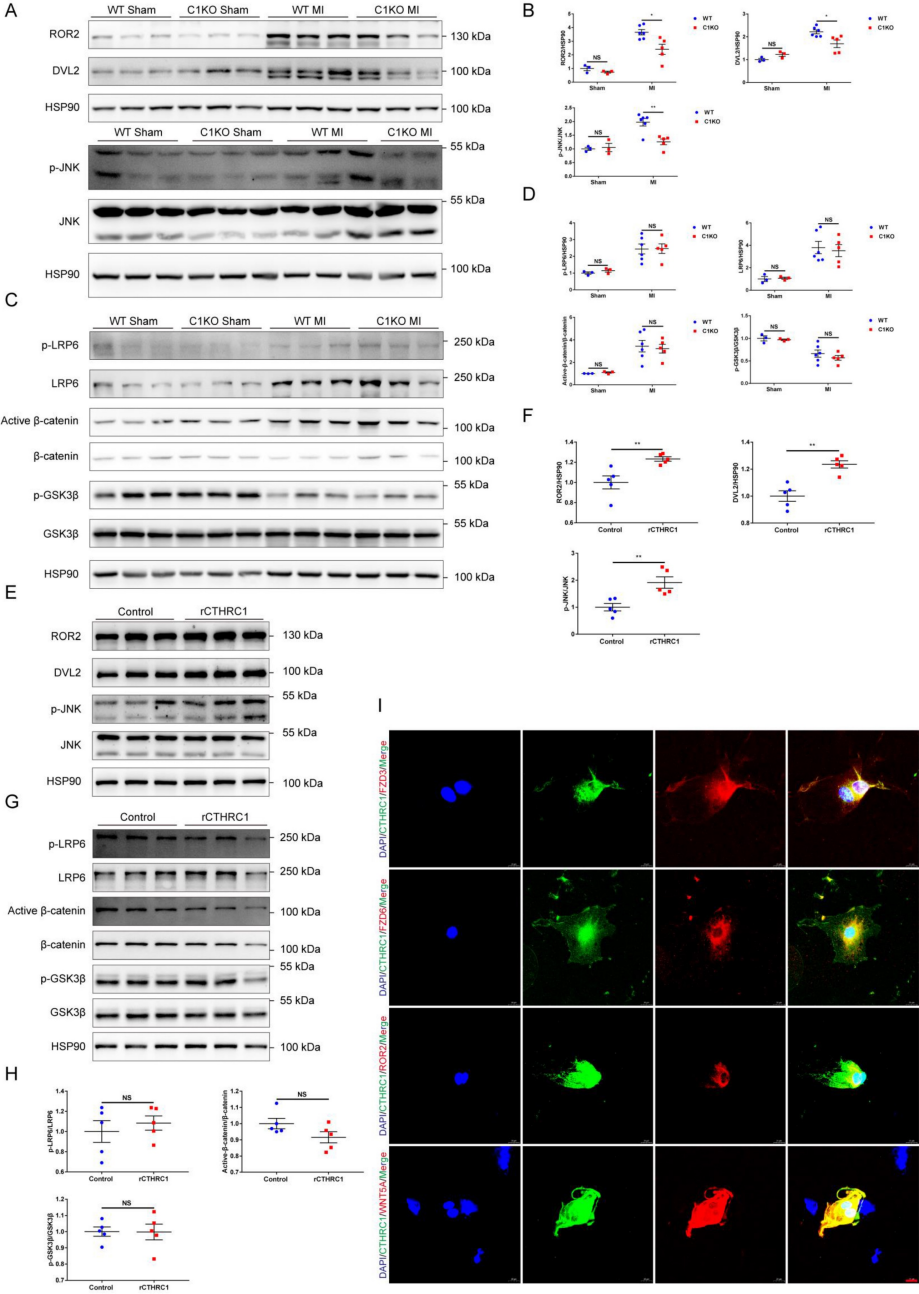
** CTHRC1 improved cardiac repair after MI via selectively activating non-canonical WNT5A-PCP signaling pathway**. **(A-D)** Western blot analyses for the indicated proteins of non-canonical WNT5A-PCP signaling axis **(A and B)** and canonical WNT3A-β-catenin signaling pathway **(C and D)** extracted from the infarcted heart tissues of WT and C1KO mice at day 7 after MI (n=3-6).** (E-H)** Western blotting of primary cardiac fibroblasts treated with rCTHRC1 (1 μg/ml) for 1h to detected the protein levels of non-canonical WNT5A-PCP signaling axis **(E and F)** and canonical WNT3A-β-catenin signaling pathway **(G and H)** (n=5). **(I)** Immunofluorescence co-staining for CTHRC1 with FZD3, FZD6, ROR2, or WNT5A in primary cardiac fibroblasts followed by treatment with TGFβ1 (10 ng/ml) for 24 h (bar=20 μm). Data are presented as mean±SEM. *P<0.05. **P<0.01. ***P<0.001. NS: not significant. Data were compared using two-way ANOVA followed by Bonferroni multiple comparison test **(B and D)** and unpaired two-tailed Student's t test **(F and H)**.

In order to further elucidate the molecular mechanisms underlying the effects of CTHRC1 on post-infarction wound repair, the transcriptomes of WT and C1KO left ventricle tissues at 7 days after MI were examined using RNA sequencing. Bioinformatic analysis demonstrated that Cthrc1 deficiency significantly changed the expression of genes in MI hearts (Figure **S6A** and **S6B**). Further KEGG analysis revealed the top 20 enriched KEGG pathways of the DEGs with Cthrc1 deficiency (Figure **S6C**) and several DEGs were selected based on the RNA-seq data (Figure **S6D**). Among them, FZD6 is regarded as the receptor of WNT signaling pathway, which has been reported to be involved in the development of myocardial infarction 45-53. To confirm these results. We also used online available databases STRING and GeneMANIA to predict the protein-protein interactions. The prediction results (Figure **S7**) revealed that CTHRC1 could interact with ROR2, WNT5A, FZD3, and FZD6, all of which participate in the non-canonical WNT5A-planar cell polarity (PCP) signaling pathway, which is a known pathway of developmental processes. Thereby, we analyzed the key signals and target genes downstream of non-canonical WNT5A-PCP signaling pathway. The expressions of its target genes, such as ROR2, DVL2, and p-JNK/JNK were differently expressed between C1KO mice and WT mice post-MI (Figure **7A** and** 7B**). It has been reported that FZD3 and FZD6 were also involved in the canonical WNT3A-β-catenin signaling axis. Thus, to bear out the impacts of CTHRC1 on canonical WNT3A-β-catenin signaling pathway, its target proteins such as p-GSK3β/GSK3β, active-β-catenin/β-catenin, p-LRP6 and LRP6 were also detected by immunoblotting. Actually, these proteins were not affected in the absence of Cthrc1 (Figure **7C** and** 7D**). Furthermore, *in vitro*, the isolated primary cardiac fibroblasts were treated with rCTHRC1 protein. In contrast, the expression levels of ROR2, DVL2, and p-JNK/JNK were increased in comparison with that of the untreated cardiac fibroblasts (Figure **7E** and** 7F**). Consistent with the *in vivo* experiments, its downstream proteins of the canonical WNT3A-β-catenin signaling pathway still remained unchanged with the stimulation of rCTHRC1 protein (Figure **7G** and** 7H**). Based on the above results, we focused on the role of non-canonical WNT5A signaling pathway in post-infarction wound healing. Previous study has borne out that CTHRC1 was a WNT co-factor that selectively activates the non-canonical WNT5A-PCP signaling pathway by forming a stabilized CTHRC1-WNT5A-FZD3/6-ROR2 complex to enhance the interaction of WNT5A with FZD3/6-ROR2^16^. To verify whether CTHRC1 mediates its effects on WNT5A-FZD3/6-ROR2 via a direct interaction, we performed an immunofluorescence co-staining in isolated primary cardiac fibroblasts treated with TGFβ1. The results revealed that CTHRC1 and WNT5A, ROR2, as well as FZD3/6 co-localized in cardiac fibroblasts, respectively (Figure **7I**). To sum up, these results demonstrate that CTHRC1 interacts with WNT5A, ROR2, and FZD3/6 to selectively activates non-canonical WNT5A signaling pathway in cardiac fibroblasts, subsequently promoting cardiac fibroblast activation and improving wound healing after MI.

**Figure 8:**
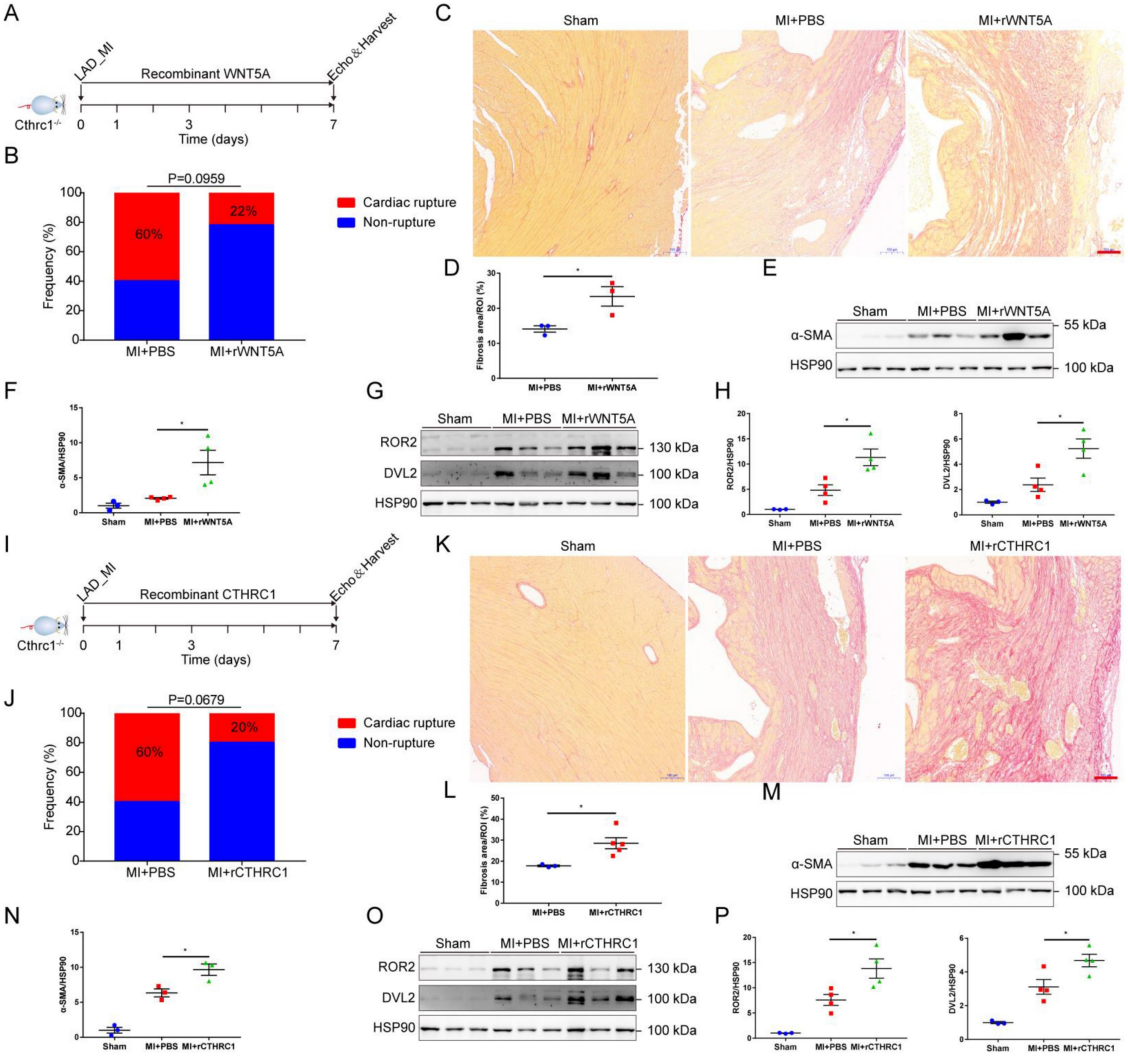
** Cthrc1 loss-induced cardiac rupture after MI was partly reversed by rWNT5A or rCTHRC1 protein**.** (A)** Timeline of the experimental design for **Figure 8B-8H**.** (B)** Total cardiac rupture frequency in C1KO mice with PBS (n=10) and with rWNT5A (n=9) at day 7 post-MI. **(C)** Picrosirius red staining performed on heart cross sections to examine collagen density in the scar of C1KO mice with PBS and with rWNT5A at day 7 after MI (bar=100 μm). **(D)** Quantitative analysis of fibrotic areas at day 7 after MI in C1KO and rWNT5A-treated mice (n=3). **(E and F)** α-SMA expression was analyzed by western blotting in the infarct zone of C1KO hearts at day 7 post-MI (n=3-4). **(G and H)** Western blot analyses for the indicated proteins of non-canonical WNT5A-PCP signaling axis in the infarct area of C1KO hearts at day 7 post-MI (n=3-4). **(I)** Timeline of the experimental design for **Figure 8J-8P**.** (J)** Total cardiac rupture frequency in C1KO mice with PBS (n=10) and with rCTHRC1 (n=10) at day 7 post-MI. **(K)** Picrosirius red staining performed on heart cross sections to examine collagen deposition in the scar of C1KO mice with PBS and with rCTHRC1 at day 7 post-MI (bar=100 μm). **(L)** Quantification of fibrotic areas at day 7 after MI in C1KO and rCTHRC1-treated mice (n=3-5). **(M and N)** α-SMA expression was detected by immunoblotting in the infarct zone of C1KO hearts at day 7 post-MI (n=3). **(O and P)** Western blot analyses for the indicated proteins of non-canonical WNT5A-PCP signaling axis in the infarct area of C1KO hearts at day 7 after MI (n=3-4). Data are presented as mean±SEM. *P<0.05. **P<0.01. Data in **B** and **J** were compared by χ^2^ test. Data were analyzed using unpaired two-tailed Student's t test **(D and L)** and one-way ANOVA followed by Bonferroni multiple comparison test **(F, H, N, and P)**.

To further confirm that CTHRC1 improved post-MI wound healing partly by non-canonical WNT5A signaling pathway, rWNT5A protein was continuously injected into C1KO mice for 7 days (0.1 μg/d) after MI (Figure **8A**). In agreement with our expectations, rWNT5A protein injection had lower frequency of cardiac rupture compared with PBS control groups (Figure **8B**). Interestingly, rWNT5A protein therapy promoted more collagen-rich scar formation than those in C1KO mice treated with PBS (Figure **8C** and** 8D**), as was also evidenced by the increased α-SMA expression in the infarct area of C1KO hearts at day 7 after MI (Figure **8E** and** 8F**). Furthermore, the expression of downstream proteins involved in non-canonical WNT5A-PCP signaling pathway was up-regulated caused by rWNT5A protein injection (Figure **8G** and** 8H**). Taken together, rWNT5A protein treatment reversed Cthrc1 loss-induced post-MI cardiac rupture, which further supports that CTHRC1 improved post-MI cardiac repair partly by non-canonical WNT5A signaling pathway.

We next tested CTHRC1 as a therapy for acute MI. To assess the therapeutic potential of CTHRC1 in the early phase of acute MI, we induced MI by coronary artery ligation followed by a continuous intraperitoneal injection of rCTHRC1 protein for 7 days (1 μg/d) (Figure **8I**). Intriguingly, rCTHRC1 protein therapy could prevent cardiac rupture after MI in C1KO mice (Figure **8J**). Moreover, C1KO mice treated with CTHRC1 developed bigger infarct scars compared with PBS control group (Figure **8K** and** 8L**). In line with this, rCTHRC1 protein injection increased the α-SMA expression in the infarct zone of C1KO post-MI hearts (Figure **8M** and** 8N**). Similarly, rCTHRC1 protein treatment up-regulated downstream proteins of non-canonical WNT5A-PCP signaling pathway (Figure **8O** and** 8P**). All those above results suggest that rCTHRC1 protein may represent a promising novel therapeutic agent in the early phase of acute MI.

## Discussion

In the absence of ischemic injury, cardiac fibroblasts remain quiescent but play an important part in maintaining extracellular matrix network. During the proliferative phase of cardiac repair, cardiac fibroblasts migrate and undergo myofibroblast transdifferentiation as well as express α-SMA and secret extracellular matrix proteins in abundance to maintain the structural integrity of the post-infarction ventricle 3. CTHRC1 is one such extracellular matrix protein with a short collagen triple helix repeat domain, which was initially identified in screening for differentially expressed genes in balloon-injured versus normal arteries and involved in vascular remodeling 7-9. Here, we demonstrated a crucial role of CTHRC1 in post-MI cardiac repair. In the present study, we reported that, first, serum levels of CTHRC1 were elevated in MI mice and CTHRC1 expression was increased in cardiac fibroblasts after MI; second, TGFβ1 induced CTHRC1 expression in cardiac fibroblasts via canonical TGFβ1-Smad2/3 signaling pathway; third, CTHRC1 improved wound healing and promoted cardiac fibroblast activation *in vitro*; fourth, Cthrc1 deficiency aggravated cardiac function and exacerbated MI-induced cardiac rupture as well as reduced collagen-rich scar formation after MI; fifth, CTHRC1 improved cardiac repair after MI via selectively activating non-canonical WNT5A-PCP signaling pathway; finally, Cthrc1 loss-induced cardiac rupture after MI was partly reversed by rWNT5A or rCTHRC1 protein. Together, these findings reveal the protective function of CTHRC1 in MI setting and provide novel insights into the molecular mechanisms underlying the wound healing after MI.

The heart contains stores of latent TGF-β1 that can be rapidly activated following MI 54. Active TGFβ1 binds to TGFβ receptors I/II, further promoting the phosphorylation of Smad2/3. Then activated Smad2/3 integrates Smad4 into it, which enables this complex to translocate into the cell nucleus and further transcribe fibrosis-related genes 41. Our study identified CTHRC1 as a key factor for cell-specific canonical TGFβ1-Smad2/3 signaling axis in the heart. We revealed that TGFβ1 induced CTHRC1 expression in nonmyocyte cells (mainly in cardiac fibroblasts). However, the expression of CTHRC1 was only mild in endothelial cells and not at all in cardiomyocytes.

In this study, CTHRC1 released from cardiac fibroblasts could promote cardiac fibroblast migration and activation, subsequently boosting scar formation as well as enhancing wound repair post-MI. In agreement with our observations, CTHRC1 producing from lung fibroblasts also contributed to the activation of fibroblasts in the lung 33. As of now COVID-19 still in tough situation. Intriguingly, researchers have dissected that expansion of CTHRC1^+^ pathological lung fibroblasts resulted in rapidly progressing pulmonary fibrosis in patients with COVID-19^55^, indicating that CTHRC1 might be identified as a novel potential therapeutic target for COVID19 in the future. Of course, CTHRC1 is also regarded as a “selfless” protein that CTHRC1 acted on other cell populations such as osteoblasts, macrophages, and endothelial cells to participate in tissue reparative response. For instance, CTHRC1 produced by osteoclasts might function as a novel guidance molecule to recruit stromal or osteogenic cells into bone resorption sites, thereby facilitating the bone formation activity 12. Previous study conducted by our colleagues found that CTHRC1 increased M2 macrophage via activating the TGF-β and Notch signaling pathways to further improve acute wound healing in a polyvinylalcohol sponge implantation mouse model 56. The latest interesting study published in *ATVB* certified that the healthy (young) aortic valve was sufficient to repair mild endothelial injury through restoration of endothelium barrier function in short term, which was mediated by TGFβ1-CTHRC1 signaling pathway 57.

Using a permanent mouse model of acute MI, we further illuminated a protective role for cardiac fibroblast-derived CTHRC1 in post-infarction wound repair. Confirming this was the fact that CTHRC1 deficiency increased post-MI cardiac rupture by up-regulating the expression of MMPs and preventing cardiac fibroblast activation as well as reducing collagen deposition. The protein levels of collagen I and collagen III as well as α-SMA were down-regulated in MI C1KO mice, suggesting a lower level of myofibroblast transformation in C1KO mice. As is known to all, activated myofibroblasts have been recognized as the major matrix-synthetic cells in the ischemic heart. They can migrate into the damaged tissue post-MI and express α-SMA as well as produce a great mass of extracellular matrix proteins, thereby resulting in collagen-rich scar formation to prevent cardiac rupture. Moreover, post-MI angiogenesis plays significant roles in the granulation tissue formation 3. As expected, Cthrc1 deficiency also affected angiogenesis after MI, as evidence by lower CD31^+^ endothelial cells and the reduced protein levels of CD31 in C1KO mice. To my relief, two recent studies have also identified CTHRC1 as consistently increased with MI in mice through a great quantity of gene expression datasets 58, 59. During the course of our study, using single-cell RNA sequencing, CTHRC1 was also bioinformatically identified as a critical cardiac fibroblast-derived secreted factor following ischemic cardiac injury potentially affecting cardiac fibroblast migration *in vitro*. Interestingly, Cthrc1 deficiency led to the obvious lethality owing to cardiac rupture in post-MI mice 40. However, the underlying molecular mechanisms for the momentous role of CTHRC1 in cardiac repair post-MI remains elusive.

The WNT signaling pathways consist of canonical WNT3A-β-catenin signaling pathway and non-canonical WNT5A-Ca^2+^ signaling pathway as well as non-canonical WNT5A-PCP signaling pathway. After WNT5A binds to FZD3/6-ROR2, the DSH-Rac as well as DSH-Rho complexes activate JNK to modulate the PCP signaling pathway 60. WNT5A signaling pathway has been demonstrated to mediate cardiac fibroblast migration and activation 45, 46, 52. It has been reported that CTHRC1 positively activated the non-canonical WNT5A-PCP signaling pathway by binding directly to FZD3/6 other than canonical WNT3A-β-catenin signaling axis in the inner ear and hair follicle development 16, 18. To dissect the underlying molecular mechanisms with regard to the effects of CTHRC1 on cardiac repair post-MI, RNA sequencing of WT and C1KO left ventricle tissues 7 days after MI was performed to screen the differently expressed genes. Besides, the predicted functional associations between proteins obtained from the online available databases STRING and GeneMANIA revealed that CTHRC1 could interact with ROR2, WNT5A, FZD3, and FZD6, which was further confirmed in our study by immunofluorescence co-localization. Moreover, using a permanent mouse model of acute MI and ex vivo isolated primary cardiac fibroblasts treated with rCTHRC1 protein, we observed the reduced expression of downstream proteins involved in non-canonical WNT5A-PCP signaling pathway in C1KO mice, whereas the levels of these proteins were up-regulated in the isolated primary cardiac fibroblasts from WT mice, which was stimulated with rCTHRC1 protein. However, the canonical WNT3A-β-catenin signaling pathway was unaffected. Importantly, rWNT5A protein treatment could reverse Cthrc1 deficiency-induced post-MI cardiac rupture, which further supports that CTHRC1 promoted post-infarction cardiac repair via selectively activating non-canonical WNT5A-PCP signaling pathway other than canonical WNT3A-β-catenin signaling axis. In accordance with our study, professor Zhang, as our sincere friend, testified that CTHRC1 participated in the progression of human colorectal cancer as well as gastrointestinal stromal tumors by promoting cancer cell migration and invasion, which was regulated by activating non-canonical WNT-PCP signaling axis but not canonical WNT-β-catenin signaling pathway 61, 62.

Translation of animal models to clinical practice is often challenging. Thereby, we firstly tested rCTHRC1 protein as a therapy for acute MI in animal models by continuous injection of rCTHRC1 protein within 7 days post-MI. Intriguingly, rCTHRC1 protein therapy could improve post-MI wound healing and reduce cardiac rupture by promoting collagen-rich scar formation, which suggests that rCTHRC1 protein may represent a promising novel therapeutic agent in the early phase of acute MI. To uncover whether CTHRC1 has a similar role in humans, we detected CTHRC1 level in peripheral blood samples obtained from patients with AMI and control group. It was gratifying that serum CTHRC1 levels were increased in patients with AMI, compared with control subjects, which implies that CTHRC1 may be regarded as a predominant mediator during AMI in humans. However, further clinical studies including large samples and reliable follow-up data should urgently be considered.

Several limitations exist in our study. Firstly, this study is limited by the lack of availability of a mouse model where Cthrc1 can be conditionally deleted specifically in cardiac fibroblasts. Up till the present moment, no large-scale effective anti-inflammatory therapeutic strategies for AMI have been successfully translated into clinical practice such as the CANTOS study, the CIRT study, and the COLCOT study. Therefore, another consideration that should be highlighted is that more work will be necessary to further define therapeutic dosing and time window for *in vivo* regulation of post-infarction wound healing by exogenous rCTHRC1 protein treatment. Moreover, our study focuses on cardiac repair in the early phase of MI, the effect of CTHRC1 on chronic ventricular remolding after MI remains to be further investigated.

In conclusion, we bear out that cardiac fibroblast-derived, canonical TGFβ1-Smad2/3-dependent extracellular matrix protein CTHRC1 promotes collagen-rich scar formation in the infarcted heart. Through direct effects on cardiac fibroblast migration and activation in the infarcted tissue, CTHRC1 enhances post-infarction wound repair and reduces cardiac rupture as well as the mortality rate via selectively activating non-canonical WNT5A-PCP signaling pathway. Further understanding of how to regulate CTHRC1 secretion from cardiac fibroblasts during ischemic cardiac injury may provide novel insights on improving wound healing in patients with AMI.

## Supplementary Material

Supplementary figures and table.

## Figures and Tables

**Figure 9:**
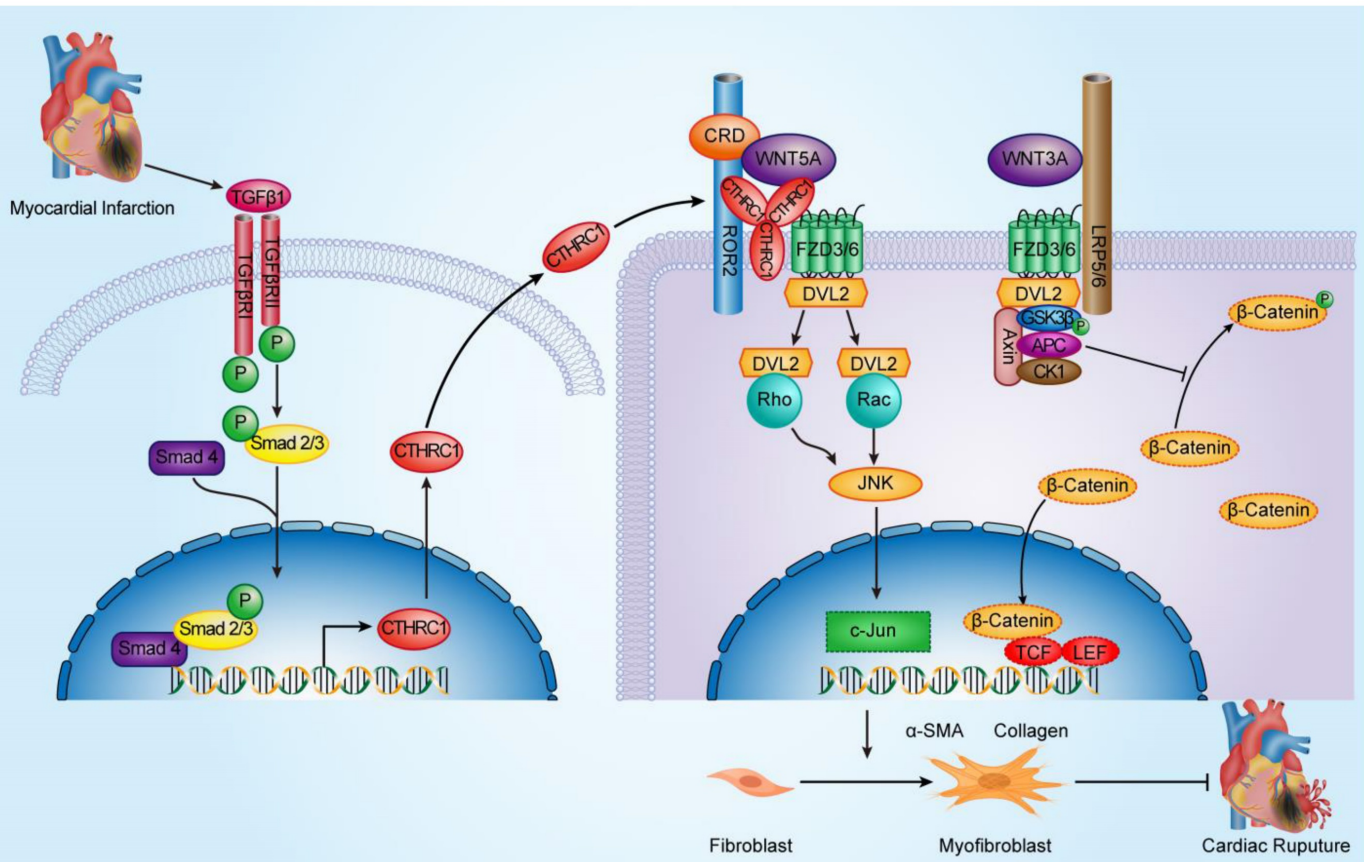
** Schematic diagram depicting the role of CTHRC1 in cardiac repair after MI.** TGFβ1 can be rapidly activated following MI. Active TGFβ1 binds to TGFβ receptors I/II, further promoting the phosphorylation of Smad2/3. Smad4 then binds to activated Smad2/3, which enables this complex to translocate to the nucleus and transcribe Cthrc1 genes. CTHRC1 secreted by cardiac fibroblasts after intracellular synthesis and glycosylation modification acts on other adjacent cardiac fibroblasts in a paracrine manner. CTHRC1 selectively activates the non-canonical WNT5A-PCP signaling pathway by forming a stabilized CTHRC1-WNT5A-FZD3/6-ROR2 complex to enhance the interaction of WNT5A with FZD3/6-ROR2, further boosting the migration and activation of cardiac fibroblasts in the infarcted tissue post-MI and increasing collagen-rich scar formation after MI, thereby improving post-MI cardiac repair and preventing cardiac rupture.

## Abbreviations

AMI: acute myocardial infarction
α-SMA: α-smooth muscle actin
CTHRC1: collagen triple helix repeat containing 1
C1KO: Cthrc1 knock out
DEGs: differentially expressed genes
DMEM: Dulbecco's Modified Eagle's Medium
EDTA: ethylenediamine tetraacetic acid
EF: ejection fraction
ELISA: enzyme linked immune sorbent assay
FBS: fetal bovine serum
FS: fractional shortening
GEO: Gene Expression Omnibus
GO: Gene Ontology
KEGG: Kyoto Encyclopedia of Genes and Genomes
LAD: left anterior descending artery
LVEDD: left end-diastolic diameter
LVEDV: left ventricular end-diastolic volume
LVESD: left end-systolic diameter
LVESV: left end-systolic volume
MMPs: matrix metalloproteinases
PBS: phosphate-buffered saline
PCP: planar cell polarity
qRT-PCR: quantitative real-time polymerase chain reaction
rCTHRC1: recombinant human CTHRC1
rWNT5A: recombinant human WNT5A
SEM: standard error of the mean
TTC: 2,3,5-triphenyltetrazolium chloride
WT: wild type
