# Supplementary material for: Cthrc1 deficiency aggravates wound healing and promotes cardiac rupture after myocardial infarction via non-canonical WNT5A signaling pathway
Source: Int J Biol Sci. 2023 Feb 13;19(4):1299–315. doi: 10.7150/ijbs.79260 (PMC10008688; doi:10.7150/ijbs.79260)
Supplement: Supplementary file 1 — Supplementary figures and table. [file ijbsv19p1299s1.pdf]

Supplemental Material

Cthrc1 deficiency aggravates wound healing and promotes cardiac rupture after myocardial infarction via non-canonical WNT5A signaling pathway

Supplemental Materials and Methods

Mice

Cthrc1 knockout (C1KO) mice on a C57BL/6J genetic background were kindly gifted by Professor Zhigang Zhang (State Key Laboratory of Oncogenes and Related Genes, Shanghai Cancer Institute, Ren Ji Hospital, School of Medicine, Shanghai Jiao Tong University). Cthrc1 mutant mice (CDB0502K) obtained from Riken BioResource Center were generated as following: a targeting vector was constructed by inserting a cassette consisting of LacZ-pA and PGK-neopA into the first exon of Cthrc1 to replace the coding sequence with LacZ[1-3] (Figure **S8A**). Male wild-type (WT) C57BL/6J mice aged 8-10 weeks old were purchased from Beijing Vital River Laboratory Animal Technology (Beijing, China). The animals were maintained under standard temperature and humidity with free access to food and water as well as a 12-h light-dark cycle. Recombinant human CTHRC1 (rCTHRC1) protein (HY-P70092, MCE) or recombinant human WNT5A (rWNT5A) protein (ab204627, Abcam) was administered i.p. [1 μg/day for rCTHRC1 protein or 0.1 μg/day for rWNT5A protein, dissolved in 200 μl phosphate-buffered saline (PBS)] to the mice, and the control group received 200 μl PBS per day. Genotyping of C1KO mice was performed by conventional PCR (Figure **S8B**). The primer sequences used for the PCR were as follows:

Cthrc1-F: CCAGGCCTATAACAGTATGCAAGC;

Cthrc1-R-WT: CGACTCTCAGACTTACCAGGTCTAC;

Cthrc1-R-Mutant: TTGAGGGGACGACGACAGTATC.

Induction of MI

MI model was induced by a permanent ligation of the left anterior descending (LAD) coronary artery using the minimal thoracotomy approach described by Gao et al[4]. In brief, male mice aged 8-10 weeks old were anesthetized with 2% isoflurane gas inhalation using an isoflurane delivery system. A small skin cut was made over the left chest, and the LAD was ligated using a 6-0 silk suture at a site approximately equal to 3 mm from its origin. Sham-treated animals underwent an identical surgical procedure without LAD occlusion. Mice that died during surgery or died immediately after surgery as well as ejection fraction (EF)>50% within 24 h after MI were excluded from the present study. For survival analysis, each mouse found dead was subjected to postmortem examination and cardiac rupture was confirmed by the presence of a blood clot in the chest cavity and/or around the pericardium.

Echocardiography

Transthoracic echocardiography was performed with a Vevo 2100 ultrasound instrument (VisualSonics, Toronto, Canada) to evaluate cardiac function at different time points post-MI. Mice were anesthetized with 2% isoflurane gas and then fixed on a heated platform. Heart rate was maintained at approximately 450-500 bpm in all mice during the echocardiographic examination. B-mode tracings of the left ventricular endocardial border in a parasternal long axis were performed to directly assess left ventricular end-diastolic volume (LVEDV) and left ventricular end-systolic volume (LVESV). EF was calculated according to the following formula: EF=[(LVEDV-LVESV)/LVEDV]×100. M-mode tracings were conducted through the anterior and posterior left ventricular walls at papillary level in the same long axis views to measure left ventricular end-diastolic diameter (LVEDD) and left ventricular end-systolic diameter (LVESD). Fractional shortening (FS) was calculated in accordance with the following formula: FS = [(LVEDD-LVESD)/LVEDD]×100.

Histology

Heart tissue samples were harvested under anesthesia and perfused with PBS followed by fixation with 4% paraformaldehyde as well as embedded in paraffin and cut into 5 μm thick transverse sections at different levels. The sections were stained

with masson's trichrome and picrosirius red staining at apical and papillary level and then examined under ordinary polychromatic light to evaluate extent of cardiac fibrosis. The collagen volume fraction was calculated as the ratio of positive staining area to the total scar area and assessed in 10 to 15 randomly selected fields in each section. To determine the infarct size after MI, sequential cross sections of heart that were chopped above the level of the suture and 0.5 mm, 1.0 mm, 1.5 mm, as well as 2.0 mm distal to the ligature were performed with masson's trichrome staining. The infarct size was calculated as the percentage of necrotic area respect to total left ventricle area. The wall thickness of the scars at apical and papillary level was also measured. Alternatively, hearts were harvested and immediately frozen on dry ice. The frozen hearts were cut transversely into five slices of equal thickness and stained with 1% 2,3,5-triphenyltetrazolium chloride (TTC, T8877, Sigma-Aldrich) in PBS in a 37 °C water bath for 20 minutes. After fixation in 10% neutral buffered formaldehyde for 4 to 6 h, each slice was photographed. Infarct tissues stained appeared pale white, viable myocardium appeared brick red. The infarct size was expressed as the percentage of infarct area respect to total left ventricle area. These data were measured and analyzed using Image J software.

Immunohistochemistry

For immunohistochemistry analyses, the paraformaldehyde-fixed, paraffin-embedded sections of the heart tissue specimens were deparaffinized in xylene and rehydrated in a graded ethanol series. After antigen retrieval with citrate buffer (PH 6.0), blocking was performed with 3% BSA at room temperature for 30 min. A solution of 3% H_2O_2 was used to block endogenous peroxidase activity. The slices were then incubated with primary antibody against CTHRC1 (ab85739, Abcam) and vimentin (ab8978, Abcam) overnight at 4 °C. After rinsing for three times in PBS, appropriate secondary antibodies were added and incubated at room temperature for 1 h, followed by color development with DAB kit (K5007, DAKO). Images were acquired with a microscope.

Immunofluorescence

Immunofluorescence staining were performed on the paraffin-embedded sections of the heart tissue samples fixed with 4% paraformaldehyde. After deparaffinization, antigen retrieval with citrate buffer (pH 6.0), and blocking with 3% BSA at room temperature for 30 min, the sections were incubated with these antibodies overnight at 4 °C. Afterwards, the slices were washed with PBS for three times followed by incubation with appropriate fluorescent-labeled secondary antibodies for 1 h at room temperature. Nuclei were counterstained with DAPI, which was followed by visualization using a microscope. The primary antibodies used for immunofluorescence were as follows: CTHRC1 (ab85739, Abcam), vimentin (ab8978, Abcam), cTnI (ab47003, Abcam), CD31 (ab281583, Abcam).

RNA extraction and quantitative real-time polymerase chain reaction (qRT-PCR)

Total RNA was extracted from heart tissues or primary isolated cardiac cells using RNA Purification Kit (EZB-RN001-plus and B0004DP, EZBioscience) or Trizol reagent (T9424, Sigma-Aldrich), according to the manufacturer's instructions. Reverse transcription was performed with RNA to obtain cDNA with a First Strand cDNA Synthesis Kit (R222-01, Vazyme) by following conditions: 25 °C for 5 min, 50 °C for 30 min, and 85 °C for 5 min. The specific gene expression level was analyzed with a LightCycler 480 II Instrument (Roche) using SYBR green PCR mix (Q511-02, Vazyme). The relative mRNA abundance was normalized to 18S as a housekeeping gene. The qRT-PCR primers were as follows:

Cthrc1-F: CCCCAAACTATAAGCAGTGTTCG

Cthrc1-R: GGAGCGCATCTTCGTGAATGT

Collagen 1a1-F: GAAGCACGTCTGGTTTGGA

Collagen 1a1-R: ACTCGAACGGGAATCCATC

Collagen 3a1-F: CTGTAACATGGAAACTGGGGAAA

Collagen 3a1-R: CCATAGCTGAACTGAAAACCACC

18s-F: AGTCCCTGCCCTTTGTACACA

18s-R: CGTTCCGAGGGCCTCACT

Western blotting

Heart tissues or primary isolated cardiac cells were homogenized in ice-cold RIPA buffer (P0013B, Beyotime) containing protease and phosphatase inhibitor cocktail (04693132001, 04906837001, Roche). Equivalent amounts of protein were loaded into 10% or 12% SDS-PAGE and then transferred onto 0.45 μm PVDF membrane (Millipore). Membranes were blocked with 5% skimmed milk in TBST buffer for 1 h and incubated with primary antibodies with gentle rocking overnight at 4 °C. After washing with TBST three times, membranes were incubated with HRP-linked anti-rabbit IgG (7074, CST) or anti-mouse IgG antibodies (115-035-003, Jackson ImmunoResearch) for 1 h at room temperature. Finally, the protein bands were visualized using an enhanced chemiluminescence reagent (34580, Thermo Fisher Scientific) and analyzed with a luminescent image analyzer (Tanon). Quantification of the protein bands was performed with Image J software. The following primary antibodies were used in this experiment: CTHRC1 (ab85739, Abcam), MMP2 (97779, Abcam), MMP9 (38898, Abcam), CD31 (ab222783, Abcam), α-SMA (19245, CST), ROR2 (88639, CST), DVL2 (3216, CST), ROR2 (88639, CST), JNK (9252,CST), p-JNK (4668, CST), LRP6 (3395, CST), p-LRP6 (2568, CST), GSK3β(12456, CST), p-GSK3β(5558, CST), Non-p-β-catenin/Active β-catenin (19807, CST), HSP90 (4877, CST), GAPDH (60004-1-lg, Proteintech).

Enzyme linked immune sorbent assay (ELISA)

Mouse or human blood samples were collected and centrifuged at 3000rpm for 10 minutes, followed by serum collection and storage at -80 °C. The levels of CTHRC1 were measured using commercially available ELISA Kits (CSB-EL006162MO, CUSABIO), according to the manufacturer's instructions. After adding stop solution, the absorbance was measured at 450 nm using a spectrophotometer.

Adult mouse cardiomyocytes isolation

Adult cardiomyocytes were isolated from the ventricles of male WT mice using a simplified, langendorff-free method[5]. Briefly, WT mice aged 8-10 weeks were anesthetized and the chest cavity was opened to fully expose the heart. The

descending aorta was chopped and the heart was immediately flushed by injecting 7 mL ethylenediamine tetraacetic acid (EDTA) buffer into the right ventricle. The ascending aorta was clipped and the heart was transferred into a 6 cm dish containing fresh EDTA buffer. Digestion was completed through sequential injection of 10 mL EDTA buffer, 3 mL perfusion buffer, as well as 30 to 50 mL collagenase buffer [0.5 mg/ml collagenase II (LS004177, Worthington) and 0.5 mg/ml collagenase IV as well as 0.05 mg/ml protease XIV in perfusion buffer] into the left ventricle. Then the ventricles were separated and gently pulled into 1 mm pieces by forceps. Cellular dissociation was achieved using gentle trituration and digestive enzyme activity was suppressed by addition of 5 mL stop buffer. Cell suspension was filtered through a 100 μm cell strainer and cells underwent 4 sequential rounds of settling by gravity, including 1 collagenase buffer as well as 3 intermediate calcium reintroduction buffers to gradually restore calcium concentration to its physiological level. The isolated cardiomyocytes were resuspended in prewarmed plating media and plated onto a laminin (5 μg/mL, 11243217001, Thermo Fisher Scientific) precoated 6 cm dish in a humidified tissue culture incubator (37 °C, 5% CO2). After 1 hour, the isolated cardiomyocytes were harvested for further western blotting analysis.

Adult mouse primary cardiac fibroblasts isolation

5-10 hearts were harvested from WT mice or C1KO mice aged 8-10 weeks. After removing atriums and connective tissues from the hearts, the hearts were rinsed in ice-cold PBS. Then the hearts were cut into 1 mm square pieces and further digested in 1 g/L protease XIV (P5147, Sigma-Aldrich) plus 0.8 g/L collagenase IV (LS004189, Worthington) in PBS without Ca^{2+} and incubated with shaking incubator at 37 °C for 12 min/cycle (4-5 cycles). Digested hearts were filtered with a 75 μm cell strainer and centrifuged at 300 g for 10 min. The isolated cells were resuspended with 10 ml of 10% fetal bovine serum (FBS, 100-500, Gemini) in Dulbecco's Modified Eagle's Medium (DMEM, C11995500BT, Gibco) and seeded into a 10 cm dish. After 1.5 h, the medium was changed to fresh, prewarmed DMEM. Isolated primary cardiac fibroblasts were used for experiments in the 2nd passage.

Neonatal mouse primary cardiac fibroblasts isolation

Primary cardiac fibroblasts were obtained from WT neonatal mice at 1-3 days of age. The hearts were removed from the pups immediately after euthanasia and washed with ice-cold PBS. The heart tissues were cut into about 1 mm^3 and then digested with 0.125% trypsin (25200072, Gibco) in PBS at 37 °C for 20 min/cycle (3-4 cycles). After each cycle, the supernatant containing the isolated cells was transferred into a centrifuge tube and FBS was added at a final concentration of 10% to stop enzyme digestion. Afterwards, the supernatant was discarded after centrifuged at 1000 rpm for 5 min and the isolated cells were resuspended in DMEM with 10% FBS. All collected cell suspensions were passed through a 75 μm filter and plated on a 10 cm dish in 5% CO2 at 37 °C to allow fast-adherent cells (mainly primary cardiac fibroblasts) to attach. After 1.5 h, the medium was replaced with fresh, prewarmed DMEM. The 2nd passage primary cardiac fibroblasts were used for further experiments to eliminate other nonmyocytes.

Culture and treatment of mouse primary cardiac fibroblasts

The outgrown primary cardiac fibroblasts were cultured in DMEM containing 10% FBS, 100 U/mL penicillin, as well as 100 μg/mL streptomycin (10378016, Gibco) at 37 °C in the atmosphere containing 5% CO2. Before the initiation of the stimulation, the cells were starved in DMEM without FBS for 12 h. Afterward, they were treated with different concentrations of TGF-β1 (0 ng/ml, 2 ng/ml,5 ng/ml,10 ng/ml, and 20 ng/ml, 100-21, Pepro Tech) for 24 h. Also, they were stimulated by 10 ng/ml of TGF-β1 at different time points (0 h, 1 h, 3 h, 6 h, 12 h, and 24 h). Additionally, primary cardiac fibroblasts were treated with 10 ng/mL of TGF-β1 in the presence or absence of 10 μM of LY2109761 (HY-12075, MCE) or E-SIS3 (HY-13013, MCE) for 24 h. Due to the high homology between mouse Cthrc1 and human CTHRC1, 1 ug/ml of rCTHRC1 protein was used to treat mouse primary cardiac fibroblasts for 1 h or 24 h. All these cells were harvested for western blotting or immunofluorescence staining.

Wound healing assay

The migratory ability of cardiac fibroblasts was evaluated by scratch assay. Primary cardiac fibroblasts were isolated from WT mice, treated or untreated with rCTHRC1 protein. A linear wound area was created by scratching using a 200 μL pipette tip. The plate was then gently washed to remove detached primary cardiac fibroblasts. The migratory capacity of the cardiac fibroblasts was estimated as the covered area of the scratch repopulated by primary cardiac fibroblasts after 24 h.

Cell immunofluorescence staining

After stimulated with TGFβ1 or rCTHRC1 protein, primary cardiac fibroblasts growing on the glass side were fixed with 4% paraformaldehyde for 10 min at room temperature. After washing with PBS for three times, cells were permeated with 0.2% Triton X-100 in PBS for 5 min at room temperature, washed, and then blocked in 5% BSA for 1 h at room temperature. The slides were then stained with relevant primary antibodies against CTHRC1 (ab85739, Abcam), WNT5A (sc365370, SCBT), FZD3 (sc376105, SCBT), FZD6 (sc393791, SCBT), ROR2 (sc374174, SCBT), WNT3A (sc136163, SCBT) overnight at 4 °C. The following day, cell samples were incubated with Alexa Fluor-conjugated anti-rabbit IgG (A-11008, Invitrogen) or anti-mouse IgG antibodies (A-11031, Invitrogen). Nuclei were counterstained with DAPI in PBS for 1 min. Staining was visualized using a fluorescence microscope.

Clinical study participants

A total of 40 patients who presented to our hospital with AMI were consecutively enrolled in this study. The diagnosis was made based on the following three criteria: a typical history, electrocardiographic findings in conformity to Q-wave or non-Q-wave, as well as an increase in serum myocardial enzyme levels to at least 50% above the upper normal limit. They were all treated with percutaneous coronary intervention and optimal medical treatment. Besides, 40 stable patients with confirmed normal coronary artery who underwent coronary angiography were also recruited to act as a control group. Those with significant concomitant diagnosis such as cancer, serious infections, or autoimmune diseases were excluded from the present study. The baseline clinical characteristics were summarized in **Table S1.** Blood samples were

obtained from control subjects and AMI patients at day 7 after MI. This study was approved by the ethics committee of Shanghai Jiao Tong University Affiliated Sixth People's Hospital, China [Approval no. 2021-KY-108 (K)].

Data mining and bioinformatics analysis

The GEO dataset GSE95755 used in this study to screen genes that were associated with fibrosis was available at https://www.ncbi.nlm.nih.gov/gds/. We selected fibrosis-related genes that exhibited a minimum fold-change of 2 and corrected-P value <0.05 in cardiac fibroblasts post-MI. We then identified fibrosis-related genes according to the following Gene Ontology classes: GO:0044420 (extracellular matrix part), GO:0005581 (collagen), GO:0005578 (proteinaceous extracellular matrix), and GO:0031012 (extracellular matrix).

RNA sequencing and bioinformatic analysis

To investigate possible changes in transcriptome profile elicited by Cthrc1 knockout, total RNA was extracted from WT versus C1KO left ventricle tissue 7 days after MI using the Trizol Reagent (T9424, Sigma-Aldrich), according to the manufacturer's instructions. Then the concentrations and qualities of RNA samples were determined using a NanoDrop spectrophotometer. Total RNA was used to build a complementary DNA library using the VAHTS® Universal V6 RNA-seq Library Prep Kit for Illumina (NR604-02, Vazyme) according to the kit's instructions, after which the library was sequenced on a NovaSeq 6000 platform (Illumina). Afterwards, the Raw data was quality controlled with Skewer and FastQC, aligned with STAR, and counted with StringTie to the mouse genome (Mm10). DESeq2 was used to identify differential expression of genes. The genes that met the ± 2-fold change and corrected-P value <0.05 were further analyzed. Finally, the enrichment analyses of Gene Ontology (GO) and Kyoto Encyclopedia of Genes and Genomes (KEGG) were performed using topGO.

Statistical analysis

All values are presented as the mean ± standard error of the mean (SEM) of independent experiments or independent samples with given n sizes. Statistical

analysis was performed with GraphPad Prism 7.0 (Graph Pad Prism Software, Inc, San Diego, CA). The normality of data distribution was determined by Shapiro-Wilk test. For comparisons, statistical analysis was performed using unpaired two tailed Student's t test (for comparing 2 groups), one-way or two-way ANOVA (for ≥3-group comparisons with one independent variable or two independent variables) with Bonferroni multiple comparison test for normally distributed data, and Mann-Whitney *U* test (for comparing two groups) or Kruskal-Wallis test (for multi-group comparisons) for non-normally distributed data. Survival rate was evaluated using the Kaplan-Meier method and compared by log-rank test. The difference of the total cardiac rupture frequency between two groups was assessed using χ^2 test. The values identified as outlier were excluded from statistical analysis. $P < 0.05$ was considered statistically significant.

References

1. Yamamoto S, Nishimura O, Misaki K, Nishita M, Minami Y, Yonemura S, et al. Cthrc1 selectively activates the planar cell polarity pathway of wnt signaling by stabilizing the wnt-receptor complex. Dev Cell. 2008; 15: 23-36.
2. Li J, Wang Y, Ma M, Jiang S, Zhang X, Zhang Y, et al. Autocrine cthrc1 activates hepatic stellate cells and promotes liver fibrosis by activating tgf-beta signaling. EBioMedicine. 2019; 40: 43-55.
3. Zhang XL, Hu LP, Yang Q, Qin WT, Wang X, Xu CJ, et al. Cthrc1 promotes liver metastasis by reshaping infiltrated macrophages through physical interactions with tgf-beta receptors in colorectal cancer. Oncogene. 2021; 40: 3959-73.
4. Gao E, Lei YH, Shang X, Huang ZM, Zuo L, Boucher M, et al. A novel and efficient model of coronary artery ligation and myocardial infarction in the mouse. Circ Res. 2010; 107: 1445-53.
5. Ackers-Johnson M, Li PY, Holmes AP, O'Brien SM, Pavlovic D, Foo RS. A simplified, langendorff-free method for concomitant isolation of viable cardiac myocytes and nonmyocytes from the adult mouse heart. Circ Res. 2016; 119: 909-20.

Supplemental Figure legends and Table

Figure S1. Bioinformatics analysis demonstrated CTHRC1 up-regulation in cardiac fibroblasts after ischemic cardiac injury. **A** and **B,** Heat map (**A**) and volcano (**B**) plot of different gene expression patterns in cardiac fibroblasts in sham-operated hearts and post-MI hearts. **C,** Heat map of selected genes that were up-regulated in cardiac fibroblasts isolated from sham and MI mouse hearts. **D,** Single-cell sequencing revealed cardiac fibroblasts highly expressed CTHRC1 3 days post-ischemia/reperfusion. ***P<0.001.

Figure S2. Serum levels of CTHRC1 were elevated in AMI patients. Serum CTHRC1 levels of human at day 7 after MI were measured by ELISA (n=40 for control subjects and n=40 for AMI patients). **P<0.01. Data were analyzed using unpaired two-tailed Student's t-test.

Figure S3. The expression of CTHRC1 was low in endothelial cells but not in cardiomyocytes. A, Representative immunohistochemistry analyses of vimentin in both sham-operated and WT post-MI mice, including the infarct zone, the border zone, and the remote zone (bar=50 μm). **B,** CTHRC1 protein expression was determined in primary cardiomyocytes isolated from WT hearts at different time points post-MI (n=2-3). **C** and **D,** Immunofluorescence co-staining for CTHRC1 with CD31 (**C**) and cTnI (**D**) in WT hearts at day 7 post-MI (bar=50 μm).

Figure S4. Cthrc1 deficiency did not affect infarct size after MI. A, Representative photographs of TTC-stained heart sections from WT and C1KO mice at day 7 post-MI. **B,** Quantification of infarct size at day 7 after MI in WT and C1KO mice (n=3).

Figure S5. Cthrc1 deficiency did not affect collagen density in the remote area after MI. **A,** Masson's trichrome (TC) and picrosirius red (PSR) staining of heart cross sections to examine collagen deposition in the remote zones of WT and C1KO hearts day 14 after MI (bar=100 μm). **B** and **C,** Quantification of collagen volume fraction in the remote areas of WT and C1KO heart tissues at day 14 after MI (n=4). Data are expressed as mean±SEM. NS indicates not significant. Data were analyzed

using unpaired two-tailed Student's t-test.

Figure S6. RNA sequencing of WT versus C1KO left ventricle tissue 7 days after MI showed differential expression of genes. **A** and **B,** Volcano plot (**A**) and heat map (**B**) of differential expression of genes in C1KO hearts compared with WT hearts (n=3). **C,** KEGG pathway analysis of these differentially expressed genes with Cthrc1 deficiency. **D,** Selected genes differentially expressed based on the RNA-seq data.

Figure S7. The protein-protein interactions obtained from the online available databases STRING and GeneMANIA. **A,** STRING-mosue. **B,** STRING-human. **C,** GeneMANIA-mouse. **D,** GeneMANIA-human

Figure S8. Validation of Cthrc1-knockout in mice. **A,** Schematic diagram of Cthrc1 gene-targeting strategy**. B,** PCR analysis of genomic DNA from WT and C1KO mice. **C** and **D,** Western blot analyses of cardiac extracts obtained from WT and C1KO mice at day 7 post-MI and protein lysates from WT and C1KO mice primary cardiac fibroblasts followed by treatment with TGFβ1 (10 ng/ml) for 24h demonstrated that CTHRC1 protein could not be detected following disruption of Cthrc1 gene**. E,** Immunofluorescence co-staining for CTHRC1 with vimentin in C1KO heart day 7 post-MI further confirmed the validation of Cthrc1-knockout in mice (bar=50 μm).

Figure S9. Echocardiographic analysis of WT and C1KO mice at baseline. n=26-27**.** Data are expressed as mean±SEM. NS indicates not significant. Data were analyzed using unpaired two-tailed Student's t-test.

Table S1. Baseline clinical characteristics of control subjects and AMI patients.

	**Control (n=40)**	**AMI (n=40)**	**P value**
**Age**	63 ± 1.339	65.98 ± 2.035	0.2257
**Sex (male)**	18 (45%)	32 (80%)	0.0012
**Hypertension**	20 (50%)	26 (65%)	0.1748
**Diabetes mellitus**	6 (15%)	12 (30%)	0.1082
**CTHRC1 (ng/ml)**	12.39 ± 0.8796	16.83 ± 1.277	0.0053

Data are expressed as mean±SEM or n (%). Data were analyzed by unpaired two-tailed Student's t test or χ^2 test.

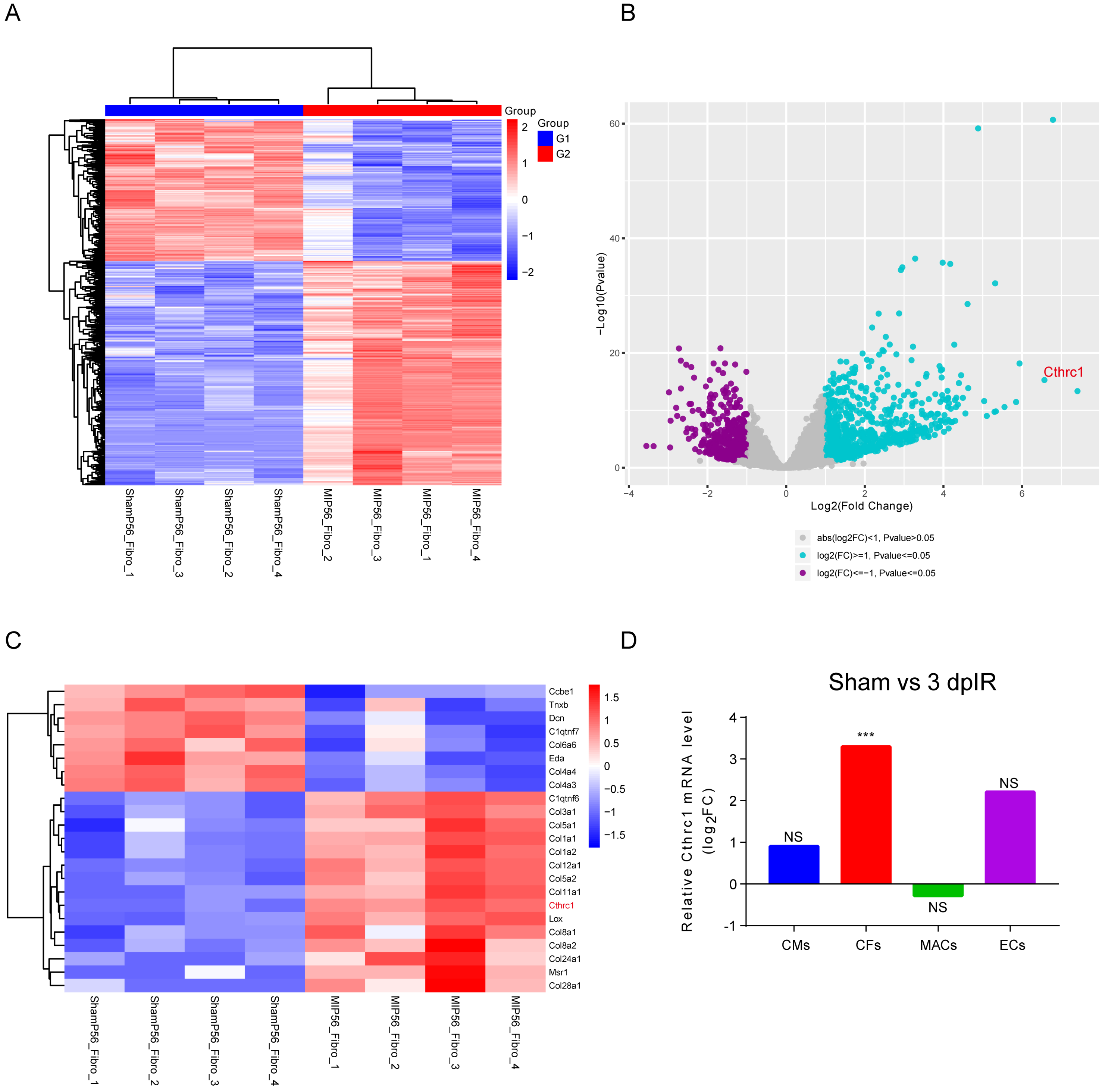

A
B
C
D
Group
G1
G2
ShamP56_Fibro_1
ShamP56_Fibro_3
ShamP56_Fibro_2
ShamP56_Fibro_4
MIP56_Fibro_2
MIP56_Fibro_3
MIP56_Fibro_1
MIP56_Fibro_4
−Log10(Pvalue)
Log2(Fold Change)
Cthrc1
abs(log2FC)<1, Pvalue>0.05
log2(FC)>=1, Pvalue<=0.05
log2(FC)<=−1, Pvalue<=0.05
Ccbe1
Tnxb
Dcn
C1qtnf7
Col6a6
Eda
Col4a4
Col4a3
C1qtnf6
Col3a1
Col5a1
Col1a1
Col1a2
Col12a1
Col5a2
Col11a1
Cthrc1
Lox
Col8a1
Col8a2
Col24a1
Msr1
Col28a1
ShamP56_Fibro_1
ShamP56_Fibro_2
ShamP56_Fibro_3
ShamP56_Fibro_4
MIP56_Fibro_1
MIP56_Fibro_2
MIP56_Fibro_3
MIP56_Fibro_4
Sham vs 3 dpIR
Relative Cthrc1 mRNA level (log2FC)
NS
***
NS
NS
CMs
CFs
MACs
ECs

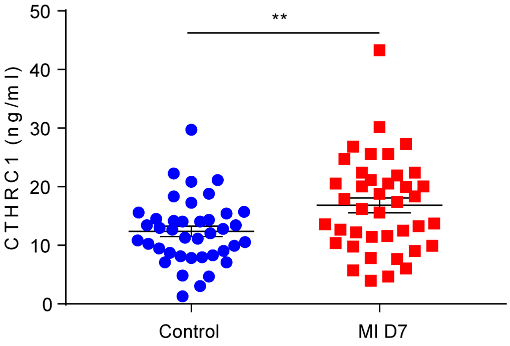

50
40
30
20
10
0
CTHRC1 (ng/ml)
**
Control
MI D7

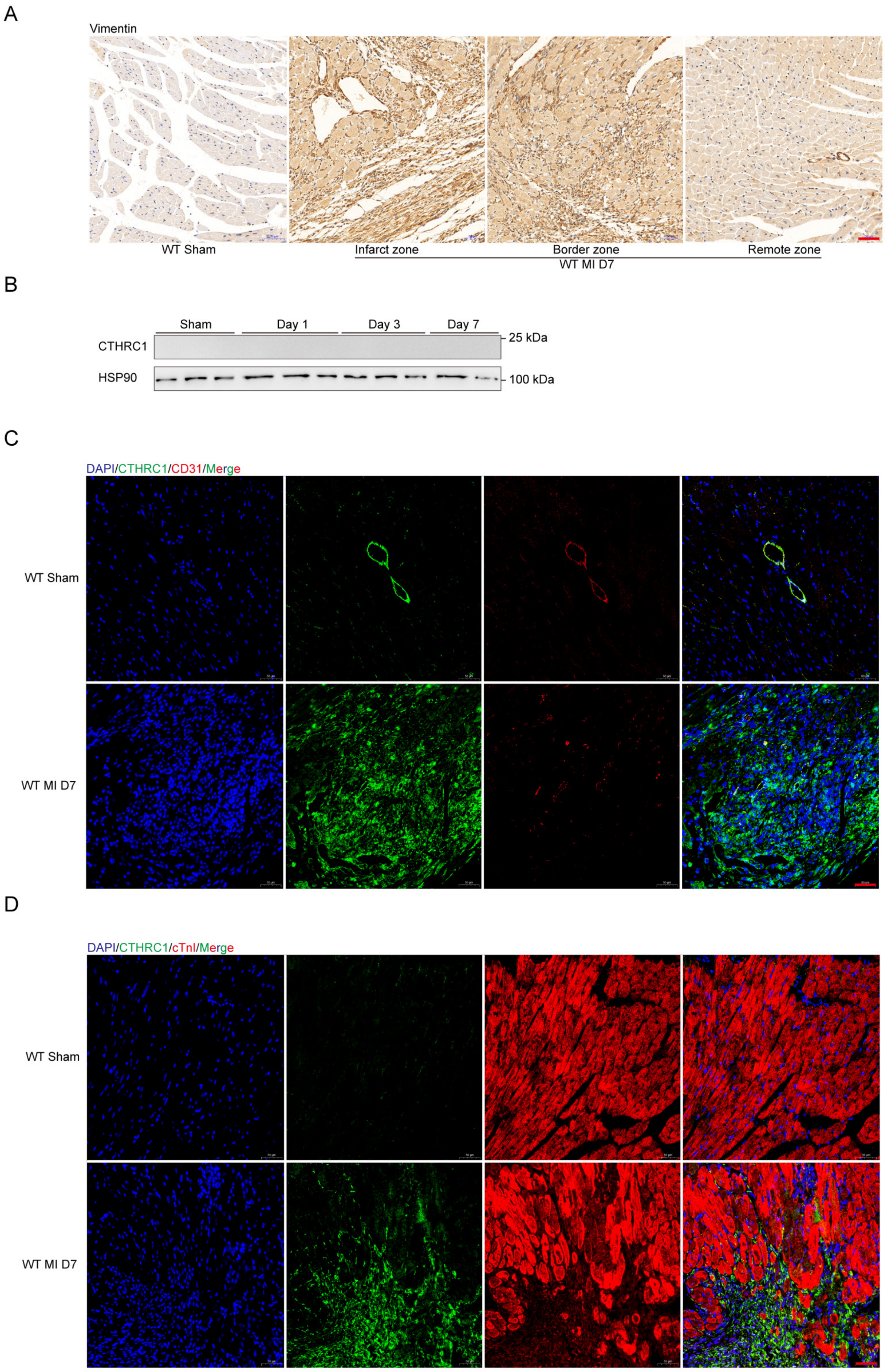
A
Vimentin
WT Sham
Infarct zone
Border zone
Remote zone
WT MI D7
B
Sham
Day 1
Day 3
Day 7
CTHRC1
HSP90
25 kDa
100 kDa
C
DAPI/CTHRC1/CD31/Merge
WT Sham
WT MI D7
D
DAPI/CTHRC1/cTnI/Merge
WT Sham
WT MI D7

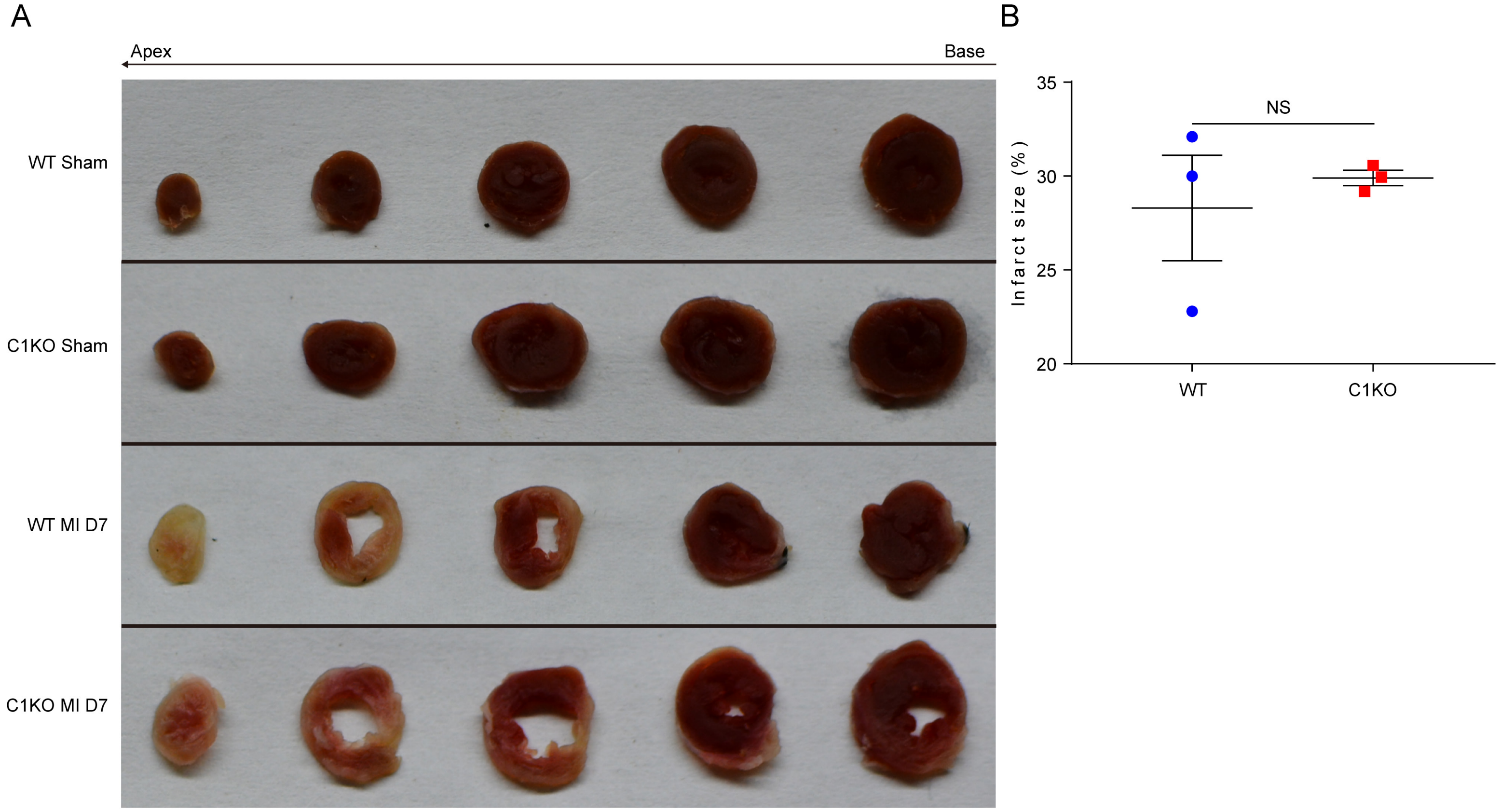
A
Apex
Base
WT Sham
C1KO Sham
WT MI D7
C1KO MI D7
B
NS
Infarct size (%)
35
30
25
20
WT
C1KO

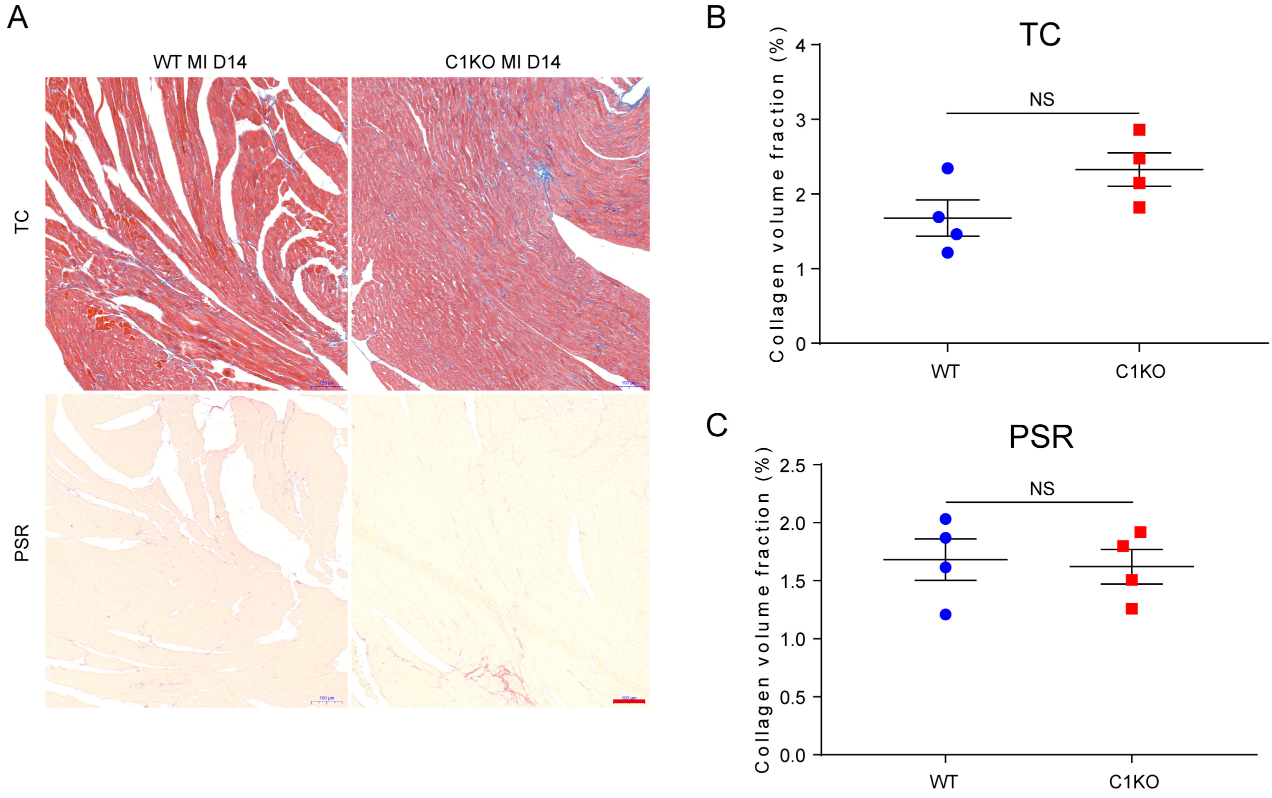
A
WT MI D14
C1KO MI D14
TC
PSR
B
TC
Collagen volume fraction (%)
NS
WT
C1KO
C
PSR
Collagen volume fraction (%)
NS
WT
C1KO

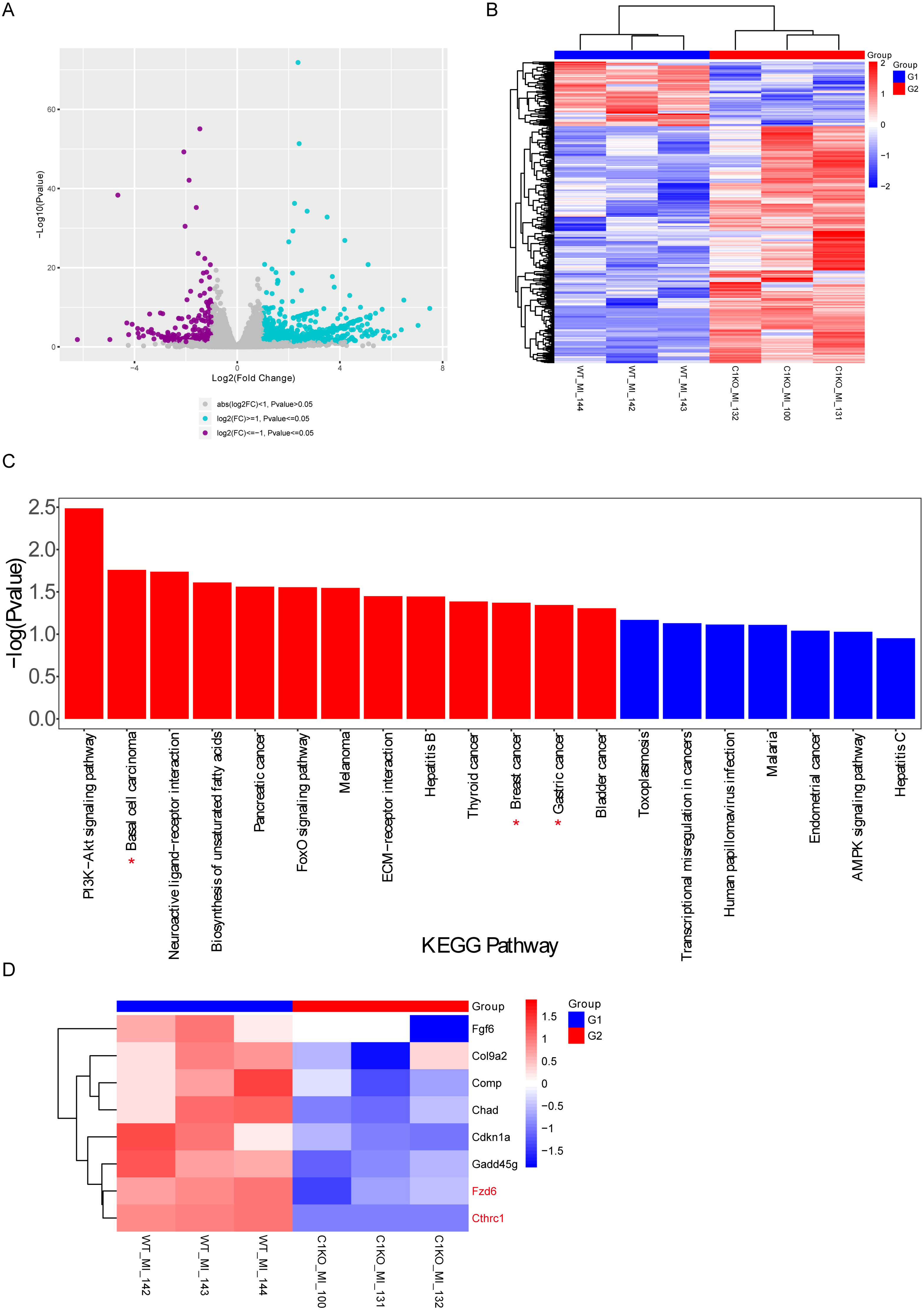

A
-Log10(Pvalue)
Log2(Fold Change)
abs(log2FC)<1, Pvalue>0.05
log2(FC)>=1, Pvalue<=0.05
log2(FC)<=−1, Pvalue<=0.05
B
Group
G1
G2
WT_MI_144
WT_MI_142
WT_MI_143
C1KO_MI_132
C1KO_MI_100
C1KO_MI_131
C
−log(Pvalue)
KEGG Pathway
PI3K−Akt signaling pathway
Basal cell carcinoma
Neuroactive ligand−receptor interaction
Biosynthesis of unsaturated fatty acids
Pancreatic cancer
FoxO signaling pathway
Melanoma
ECM−receptor interaction
Hepatitis B
Thyroid cancer
Breast cancer
Gastric cancer
Bladder cancer
Toxoplasmosis
Transcriptional misregulation in cancers
Human papillomavirus infection
Malaria
Endometrial cancer
AMPK signaling pathway
Hepatitis C
D
Group
G1
G2
Fgf6
Col9a2
Comp
Chad
Cdkn1a
Gadd45g
Fzd6
Cthrc1
WT_MI_142
WT_MI_143
WT_MI_144
C1KO_MI_100
C1KO_MI_131
C1KO_MI_132

A

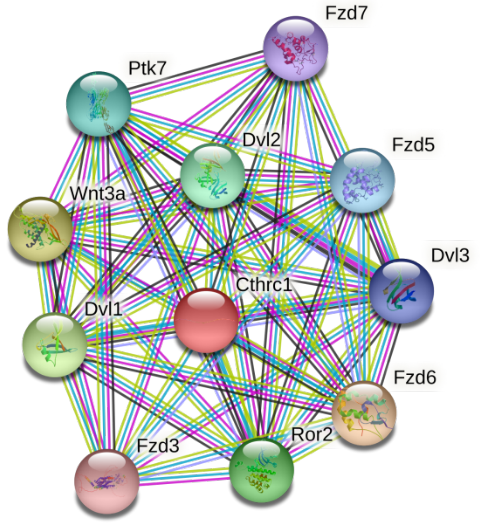
Fzd7
Ptk7
Dvl2
Fzd5
Wnt3a
Dvl3
Cthrc1
Dvl1
Fzd6
Ror2
Fzd3

B

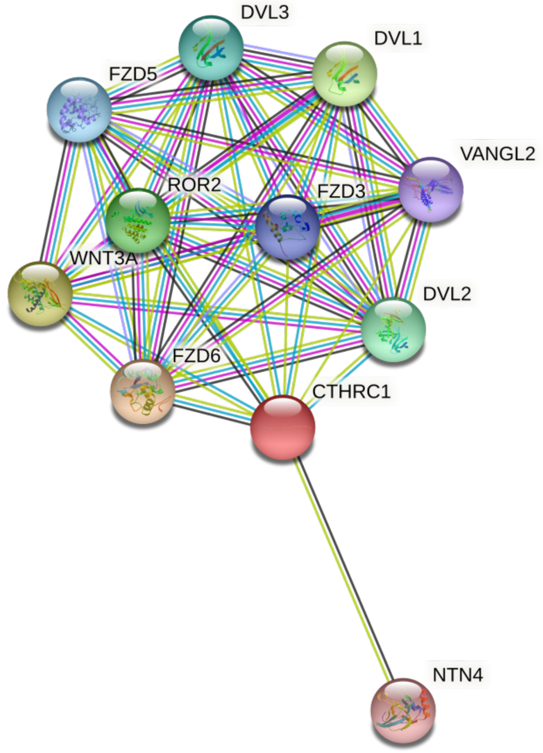
DVL3
DVL1
FZD5
VANGL2
ROR2
FZD3
WNT3A
DVL2
FZD6
CTHRC1
NTN4

C

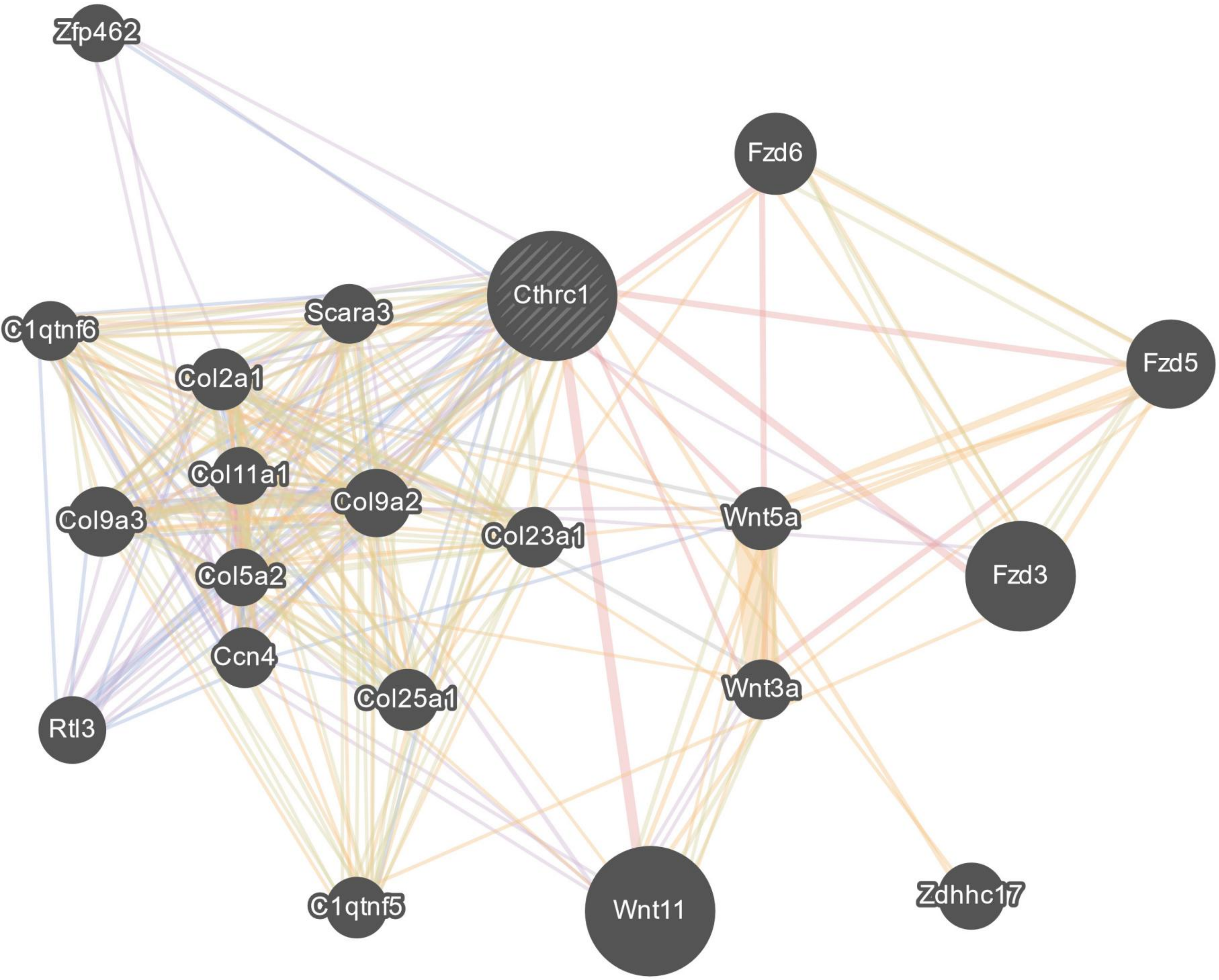
Zfp462
Fzd6
Cthrc1
Scara3
C1qtnf6
Fzd5
Col2a1
Col11a1
Col9a2
Col9a3
Col23a1
Wnt5a
Fzd3
Col5a2
Ccn4
Wnt3a
Col25a1
Rtl3
C1qtnf5
Wnt11
Zdhhc17

D

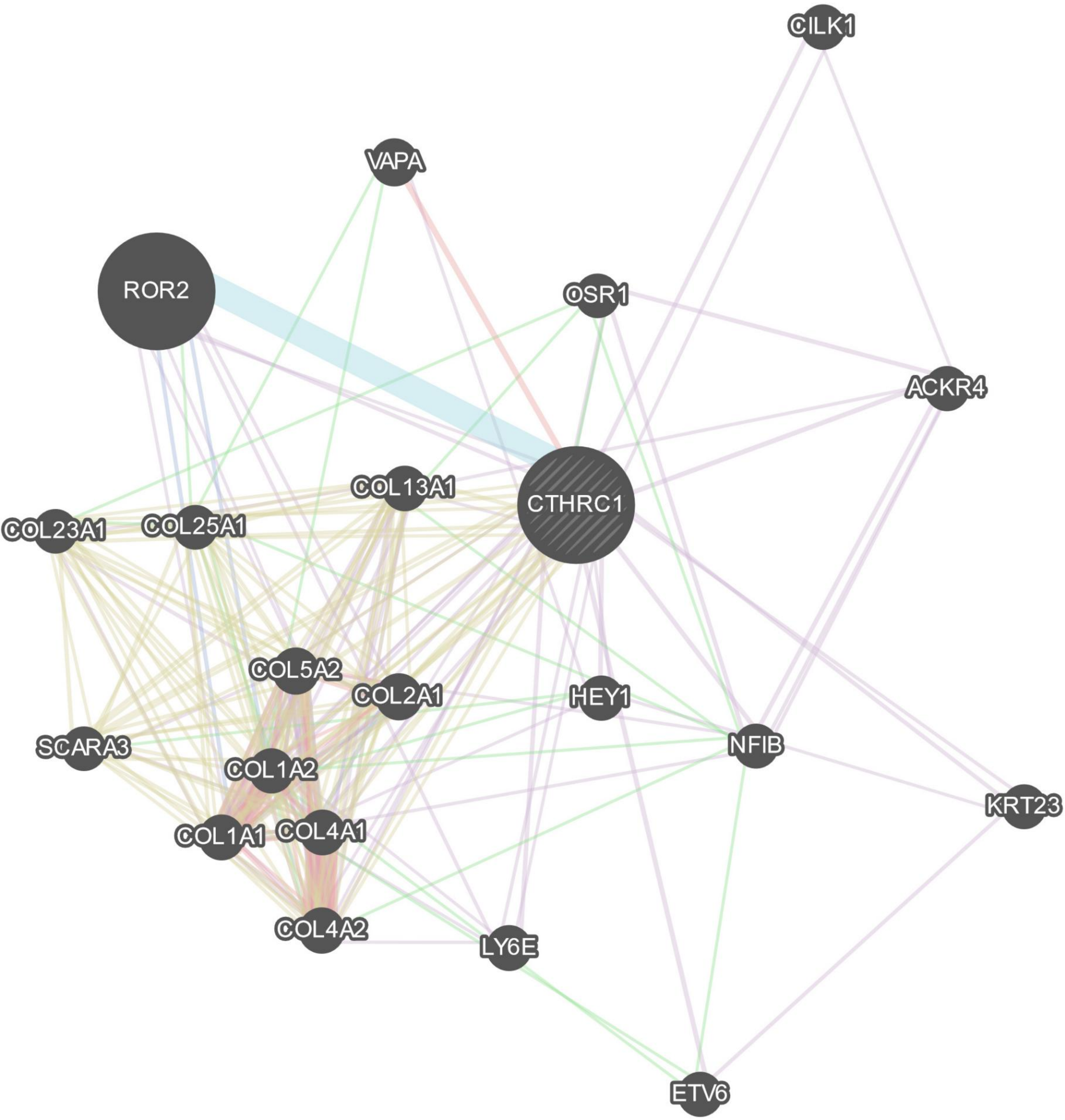

CILK1
VAPA
ROR2
OSR1
ACKR4
CTHRC1
COL13A1
COL23A1
COL25A1
COL5A2
COL2A1
HEY1
NFIB
SCARA3
COL1A2
KRT23
COL1A1
COL4A1
COL4A2
LY6E
ETV6

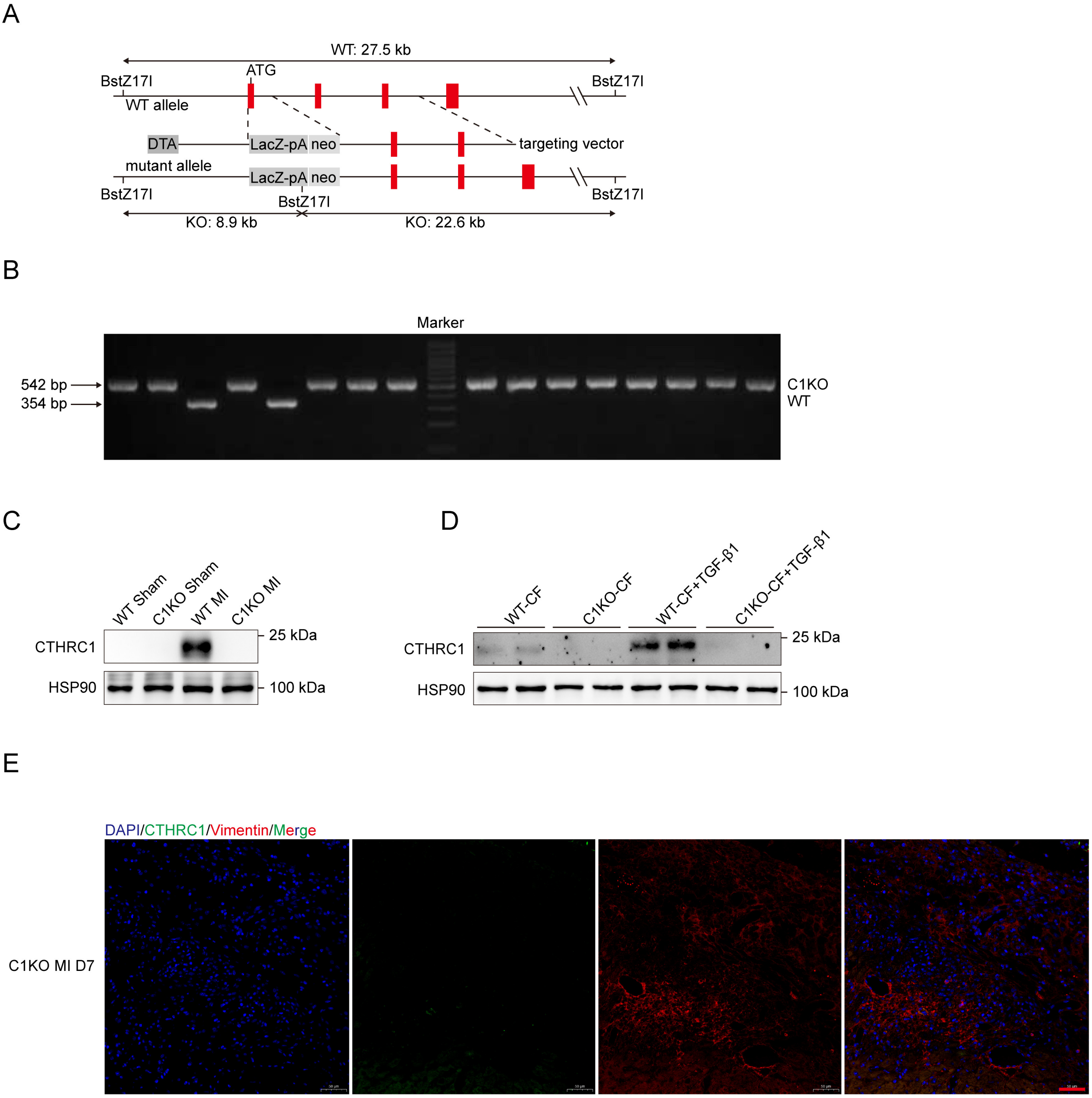

A
WT: 27.5 kb
ATG
BstZ17I
WT allele
DTA
LacZ-pA
neo
targeting vector
mutant allele
BstZ17I
KO: 8.9 kb
KO: 22.6 kb
B
Marker
542 bp
354 bp
C1KO
WT
C
WT Sham
C1KO Sham
WT MI
C1KO MI
CTHRC1
HSP90
25 kDa
100 kDa
D
WT-CF
C1KO-CF
WT-CF+TGF-β1
C1KO-CF+TGF-β1
E
DAPI/CTHRC1/Vimentin/Merge
C1KO MI D7

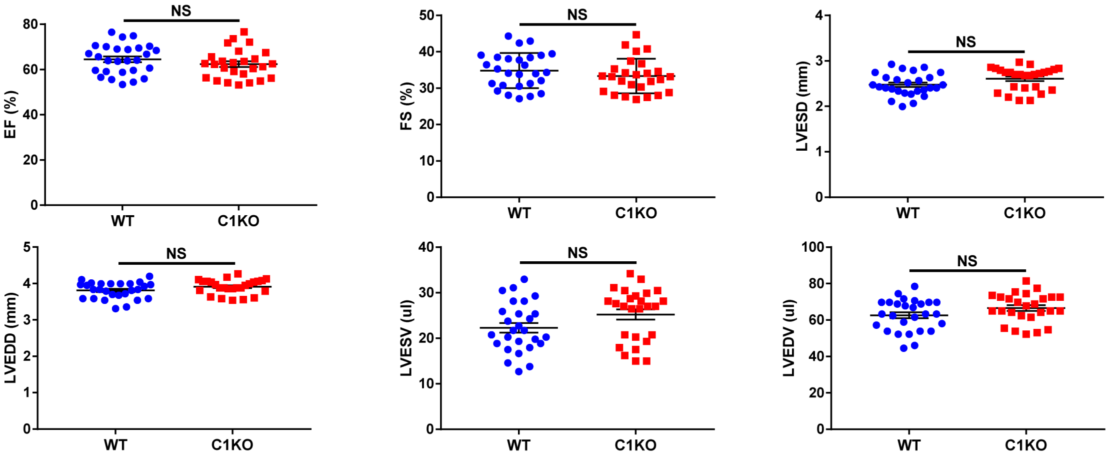

NS
EF (%)
WT
C1KO
FS (%)
LVESD (mm)
LVEDD (mm)
LVESV (ul)
LVEDV (ul)
